# The Gut Gambit: A Review of How Microbial Imbalance Fuels Metabolic Mayhem

**DOI:** 10.3390/nu18060888

**Published:** 2026-03-11

**Authors:** Lakshmayya Nunna Sai Venkata, Awdhesh Kumar Mishra, Yugal Kishore Mohanta, Sarvesh Rustagi, Ashutosh Bahuguna, Anjali Tomar, Kwang-Hyun Baek, Bishwambhar Mishra

**Affiliations:** 1 Department of Biotechnology, Chaitanya Bharathi Institute of Technology, Gandipet, Hyderabad 500075, Telangana, India; lakshmannunna@gmail.com; 2 Department of Biotechnology, Yeungnam University, Gyeongsan 38541, Republic of Korea; awdhesh@ynu.ac.kr; 3 Nano-Biotechnology and Translational Knowledge Laboratory, Department of Applied Biology, University of Science and Technology Meghalaya, Baridua, Ri-Bhoi 793101, Meghalaya, India; ykmohanta@gmail.com; 4 Centre for Herbal Pharmacology and Environmental Sustainability, Chettinad Hospital and Research Institute, Chettinad Academy of Research and Education, Kelambakkam 603103, Tamil Nadu, India; 5 Department of Food Technology, School of Agriculture, Dev Bhoomi Uttarakhand University, Dehradun 248007, Uttarakhand, India; sarveshrustagi@gmail.com; 6 Department of Food Science and Technology, Yeungnam University, Gyeongsan 38541, Republic of Korea; ashubahuguna@gmail.com; 7 School of Agricultural Sciences, K.R. Mangalam University, Gurugram 122103, Haryana, India; anjali.tomar@krmangalam.edu.in

**Keywords:** gut dysbiosis, microbiota, microbiome, oxidative stress, metabolic disorders, inflammation, epigenetics

## Abstract

Background/Objectives: An imbalance in gut microbiota, known as gut dysbiosis, results in reactive oxygen species overproduction, which can cause inflammatory conditions, damage DNA, trigger immunity, and induce epigenetic modifications of crucial genes that regulate metabolic pathways. Such a condition can also weaken the resilience of the protective gut wall and elevate colon permeability, allowing toxins from the gut to reach the liver and bloodstream, contributing to oxidative damage, autoimmune diseases, and epigenetic changes linked to metabolic disorders. Methods: The Scopus database was exclusively searched for the literature. Relevant articles were identified using predefined keywords, including gut dysbiosis, microbiota, microbiome, oxidative stress, metabolic disorders, inflammation, and epigenetics or combinations. Gut microbiota- and diet-induced metabolic disorders, particularly obesity, insulin resistance, dyslipidemia, and hypertension, may be inherited through epigenetic pathways. Results: The evidence analyzed suggests that the gut microbiota serves as a diverse metabolic and immunological organ. Its disruption affects the production of short-chain fatty acids, bile acid metabolism, immune signaling, and the redox balance, which contributes to the development of obesity, insulin resistance, and metabolic syndrome. Conclusions: This review highlights key epigenetic mechanisms underlying metabolic disorders and oxidative stress in the context of gut dysbiosis. Furthermore, therapeutic strategies targeting the gut microbiota, such as dietary interventions, prebiotics, probiotics, postbiotics, and fecal microbiota transplantation, hold promise for mitigating oxidative stress and inflammation associated with metabolic syndrome.

## 1. Introduction

The intricate human gastrointestinal system is home to a diverse and multipurpose microbial community known as the gut microbiota. It comprises a multitude of microorganisms, such as Firmicutes, Bacteroidetes, and Actinobacteria, that significantly outnumber human somatic and germ cells [1]. It is generally considered a virtual or underutilized organ that preserves a symbiotic relationship with its host [2]. Within this dynamic paradigm, the host affords a place where they can flourish, while the gut microbiota helps metabolize carbohydrates and amino acids into short-chain fatty acids, which provide the host with energy and help sustain nutrient access for the bacteria [3,4]. Furthermore, certain studies on the co-metabolism between gut microbiota and its host suggest that they circulate their unique metabolites through enterohepatic distribution, which could help conserve the fluctuating equilibrium of gastrointestinal habitats [5]. Nevertheless, different host species have distinct gut microbiota compositions. The impacts of biotic and abiotic variables, as well as internal and external influences, on gut microbiota have been documented. These elements can disrupt the gut microbiota, triggering an assortment of ailments, and impact the host’s health [6]. Regarding this issue, the spotlight has been drawn to metabolic diseases as a subset of human illnesses. Various studies over the years have provided evidence of the relationship between metabolic illnesses and gut microbiota. The consequences of sporadic changes in plant availability as food may have accelerated gut microbiome emergence in early human hunter-gatherers, supported by research on great apes. Evidently, it is enticing to believe that juxtaposing humans to apes like chimpanzees and gorillas, which share a close genetic kinship, could help acquire knowledge regarding the human gut microbiota [7]. However, the gut microbiota of baboons (ancient World monkeys) may provide insight into early human biology, as they too are clever, omnivorous, primarily terrestrial, fairly large-bodied, and socially hierarchical primates that have evolved to survive a wide range of African environments, like the savannah [8]. Though gut microbiota composition in wild baboons varies seasonally, comparative metagenomics of their gut microbiotas with those of modern humans may not reveal similarity in bacterial species-level compositions. In fact, humans no longer harbor several bacterial genera that were present when they co-diversified from apes [9]. The gut microbiotas of animal communities developed as a result of gut physiology and typical (suggested) meals; these differences are still obvious [10]. To put it another way, the chances of microbial communities identifiable as being typical of the gut of a primate or another animal to coevolving is prominent. It is possible to identify the evolutionary history of certain contemporary microbiotas groups and of humans through genetic comparisons. *H. pylori* serves as the most obvious illustration of genetic variety in connection to human migration [11]. The most significant association between the host and host-associated microbial populations is found in humans. To dominate other microorganisms and nurture particular microbial ecosystems throughout the intestine, the resident bacteria that populate these regions have developed unique processes to acquire the nutrients provided and use them as efficiently as possible.

### 1.1. Microbial Metabolism and Host Metabolic Homeostasis

Since many host–microbiota interactions are based on metabolites they are significantly impacted by microbial metabolism and nutrient availability under normal and diseased contexts. For instance, under ideal circumstances, the colon is anaerobic and provides an environment conducive to the growth of obligate anaerobic microbes that obtain energy by substrate-level phosphorylation [12]. A feedback cycle connecting host and microbial metabolisms is established when colonocytes receive signals from microbially generated butyrate, a short-chain fatty acid (SCFA), to perform β-oxidation. Proteobacteria and other facultative anaerobic bacteria are minor constituents of the vast gut microbiota owing to restricted oxygen availability [13]. The ileum absorbs a bulk of the bile salts, whereas the microbiota in the large intestine metabolize the residual primary bile salts [14]. It is widely acknowledged that gut ecosystems regulate metabolic homeostasis. Given that metabolic syndromes and the microbiota are closely associated, some recent studies have sought to describe the microbiota’s function in the pathophysiology of such disorders. Compared with healthy individuals, patients with metabolic syndromes likely have an altered microbial ecosystem (dysbiosis), although the precise microbial composition is unknown [15]. Such a diverse environment, which includes several bacteria-derived metabolites, may control immunity, inflammation, and metabolic homeostasis. The impact of the microbiome on metabolism has only recently been recognized, and the details are still unclear. Modulation of insulin signaling, hepatic gluconeogenesis, circadian host biology, and the uptake of epithelial lipids by the microbiota are few potential theories for this regulation, which most likely function in harmony [16]. Microbiome-based medical conditions rely on full metagenomic DNA sequencing. Discovering communities is only one aspect of microbiome comprehension; the next step is ascertaining the potential impacts of metabolic capacity on microbial performance, such as the host–microbiome relationship [17]; however, this phantom system has been ignored. The link between gut microbiota and host health and disorders is being examined not only in contemporary medicine but also in conventional medicine, such as Chinese and Indian medicine, and Ayurveda. Contemporary medicine adopts an integrated strategy—an interaction between the organs and spirit—usually followed in traditional medicine. A majority of these traditional systems prioritize diet [18].

### 1.2. Integrative Mechanistic Framework Linking Dysbiosis to Metabolic Disease

**Figure 1:**
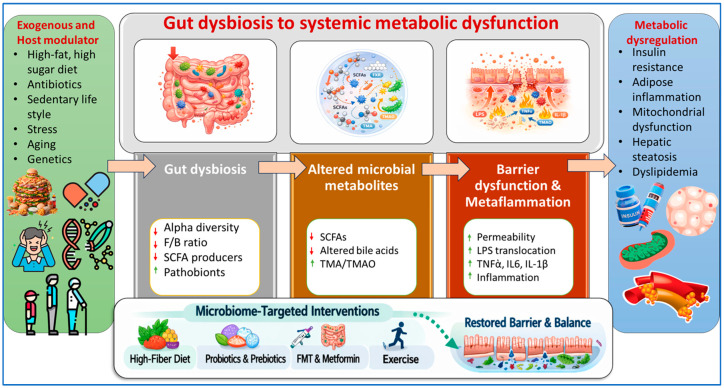
Integrative mechanistic framework linking gut dysbiosis to systemic metabolic dysfunction. Arrows indicate the progression of events from exogenous and host-related factors to gut dysbiosis, altered microbial metabolites, barrier dysfunction, and metabolic dysregulation. Upward (↑) and downward (↓) arrows denote increase and decrease, respectively.

The gut microbiota encompasses the gut microbes and their metabolites, and they affect the immune and metabolic systems of the host (e.g., systemically). An overview of the proposed integrative mechanistic framework connecting gut dysbiosis with systemic metabolic dysfunction is presented in Figure 1.

Myriad imbalances in the gut microbiota impair the production of short-chain fatty acids, disrupt bile acid metabolism, and make the gut more permeable than it should be, which results in the transfer of endotoxins to the inner layer of the gut [19]. This process activates the immune system and causes chronic low-grade or systemic inflammation (metaflammation). Metaflammation causes a reduction in insulin sensitivity, alterations in the function of adipose tissue (e.g., increases the amount of fat), causes ectopic fat storage in the liver, alters the functioning of the mitochondria, and increases the risk of developing obesity, type 2 diabetes and cardiometabolic disease. The gut microbiota in the host systemically functions as a trigger for energy or metabolic dysregulation [20].

### 1.3. Rationale and Knowledge Gaps

According to bibliometric trends in major scientific databases, studies concerning gut dysbiosis have significantly increased over the last ten years (2015–2025). As the importance of the gut in systemic health and disease is increasingly recognized, the number of papers about “gut microbiota” and “dysbiosis” has tripled. Associations between dysbiosis and immunological dysfunction, neuroinflammation, and metabolic diseases are important areas of investigation. Even though some recent reviews have addressed gut microbiota and metabolic health, the majority remain focused on microbial composition and specific metabolites related to obesity, diabetes, or metabolic syndrome, and have not attempted to synthesize oxidative stress and the epigenetic dimensions. As an example, the most recent reviews are more inclusive and examine diversity in the gut microbiota and the composition of metabolites concerning metabolic syndrome. While they emphasize host–microbe interactions and their metabolic effects, they have almost ignored the mechanistic roles of reactive oxygen species and the associated gene regulation [19,21]. Similarly, while some reviews on the gut microbiome and obesity-associated metabolic syndrome in humans have described microbial composition and suggested therapeutic strategies, they have very little to say about the host epigenetic regulation [19]. The very few reviews that have addressed the intersection of redox balance, gut microbiota, and changes in epigenetics have focused on the integrative cross-cutting systems approach in the case of neurodegenerative disorders, and illustrate the attention being paid to the integrative approach [20].

Prior reviews have focused on the constituents of gut dysbiosis, reactive oxygen species (ROS), immune activation, and epigenetic reprogramming. In this review, we focus on the evolving correlations between these constituents and metabolic dysfunction as well as implications, such as potential transgenerational imprinting. Cross-over during such interactions is a novel aspect. The gut microbiota-derived metabolites—SCFAs, lactate, and vitamins—that modulate the immune system and resolve oxidative stress induce epigenetic effects by heterochromatin-based signaling (gene silencing) and by activating the expression of certain metabolism-associated genes of the host.

### 1.4. Methods

This review is a narrative, evidence-based synthesis of the literature available. It attempts to integrate and connect several pathways, presenting a new perspective on metabolic dysfunction instead of the conventional one based on microbial composition. It attempts to identify novel pathways and means of interventions that would enhance health. Since Scopus offers complete coverage of publications in peer-reviewed journals related to biomedicine and life sciences, it was the sole database used for the literature search. The articles published between 2000 and January 2025—focusing primarily on works published in the last 5 years—were collected to reflect current advancements in the field. They were selected based on their direct relevance to gut microbiota dysbiosis, oxidative stress, immune signaling, epigenetic regulation, and metabolic disorders. Experimental and observational reports were included to capture mechanistic and translational insights. In total, 161 papers were identified; the selected literature reflects the breadth of evidence available rather than an arbitrary subset of studies. Relevant studies were selected based on their direct relevance to gut microbiota dysbiosis, oxidative stress, immune signaling, epigenetic regulation, and metabolic disorders. Both experimental and observational studies were included to capture mechanistic and translational insights. The selected literature reflects the breadth of evidence available in this field rather than an arbitrary subset of studies.

## 2. Multifactorial Origins of Gut Microbiome Dysbiosis

Intestinal homeostasis is disrupted, and gut microbiome dysbiosis is induced by disruptions in the host–gut bacteria symbiotic equilibrium. A shift in the variety, composition, and operation of gut microbiota is referred to as gut dysbiosis. Disproportionate dietary habits, interactions with pathogens and toxins, prolonged administration of proton pump inhibitors (PPIs), usage of antibiotics, excessive alcohol consumption, a higher intake of sugar or protein, contamination with pesticides, deprived oral hygiene, and prolonged stress are a few of the factors that can cause dysbiosis [22]. The brain–gut–microbiota nexus is another pathway by which gut microbes interact with the central nervous system. The role of endocrine, neurological, and metabolic processes has been suggested, but the precise mechanisms remain unclear. These relationships can significantly affect intestinal homeostasis [23]. Atopic diseases (asthma, allergic rhinitis, and obesity), metabolic diseases (obesity, Type II diabetes mellitus [T2DM], and metabolic syndrome [MetS]), and inflammatory and autoimmune diseases (necrotizing enterocolitis, Crohn’s disease, inflammatory bowel disease [IBD], and irritable bowel syndrome [IBS]) have been connected to disruptions in the gut microbiome [24].

Broad-spectrum antibiotic exposure is a well-established driver of gut microbial disruption, particularly through depletion of commensal anaerobes and expansion of opportunistic taxa. However, they also suppress the proliferation of beneficial gut microbes, changing the microbiota composition. The gastrointestinal system is the organ most susceptible to oral antibiotics [25]. In addition to gut microbiota, antibiotic therapy harms three intestinal barriers. The intestinal epithelial cell barrier is weakened by alterations in the composition of gut microbiota, which also affects the biosynthesis of mucin, cytokines, and antimicrobial peptides (AMPs). Antibiotics that combat a wide range of pathogens are commonly utilized; these include vancomycin, macrolides, and penicillins [26]. Macrolides usually have a moderate risk for gut dysbiosis. However, a higher risk was observed with broad-spectrum β-lactams, including penicillin and cephalosporin; fluoroquinolones; clindamycin; and glycopeptides, such as vancomycin, which cause more severe and long-lasting changes.

Gut microbiota in humans and food are tightly intertwined; as human society develops, so too does the use of sugar, processed carbohydrates, and Western-style high-fat diets (HFDs). Additionally, new studies indicate that the food–microbiota interactions are becoming more individualized, emphasizing the necessity of customized modifications depending on unique situations [27]. HFDs trigger gut dysbiosis in addition to obesity. The expression of proteins involved in maintaining the integrity of intestinal tight junctions (TJs) may be inhibited by gut dysbiosis. This state is typically accompanied by an excessive growth of pathogenic bacteria, such as *Escherichia coli*, *Clostridioides difficile*, *Salmonella* spp., and *Enterococcus faecalis*, coupled with a loss of certain commensals. In addition, it can increase the production and movement of gut toxins and byproducts, including lipopolysaccharides (LPS), enterotoxins, and some secondary bile acids. Additionally, the effectiveness of the intestinal mucosal barrier is compromised by bile acid synthesis brought on by HFD. One of the primary sources of energy is carbohydrates; in contrast, the excessive intake of sugar and processed carbs may disrupt the equilibrium in intestinal microbiota. Consuming highly refined carbohydrates has been linked to a suppressed expression of genes encoding TJ proteins and elevated production of proinflammatory cytokines [28]. Drug-induced dysbiosis is medication-specific and context-dependent. While broad-spectrum antibiotics exert profound and rapid effects on microbial diversity, other drug classes such as proton pump inhibitors and certain non-steroidal anti-inflammatory drugs have been associated with compositional shifts in gut microbiota in observational studies. Conversely, several antidiabetic agents (e.g., metformin) may partially restore microbial diversity, complicating the simplistic classification of all medications as dysbiosis-inducing. Mice given a carbohydrate-rich diet exhibited alterations in gut microbiota, especially a spike in those belonging to the phylum Proteobacteria [29]. Desulfovibrionaceae were more prevalent in the ceca of the glucose-fed group, indicating a change in the microbiota composition that results in a proinflammatory profile [30].

Inflammation is the body’s natural defense against infection or injury; it is a major contributor to microbial imbalance and intestinal permeation. Gut inflammation caused by infections by pathogens, such as bacteria and viruses, can result in dysbiosis. The gut microbiota sustains a favorable consortium of symbiotic bacteria to generate an equal defense response and maintain the body’s homeostasis. Meanwhile, during dysbiosis, proinflammatory microbiota (pathogenic microorganisms) may either expand or subside. As a result, the harmony between pro- and anti-inflammatory signals alters, leading to an inflammatory phenotype that is linked to the occurrence of several illnesses, including IBD and multiple sclerosis (MS) [31]. For instance, after penetrating the mucosal layer, the intestinal epithelial cells are invaded by *Klebsiella pneumoniae*. The host macrophages are subsequently stimulated to secrete proinflammatory cytokines like tumor necrosis factor-α (TNF-α) and interleukin (IL)-1β. Such an elevated proinflammatory state disrupts intestinal homeostasis, misaligning the microbial environment [32].

Cultural diversity is a significant factor determining inter-individual variations in the fecal microbiota of 2084 people of different ethnicities who inhabit the same cities. In this case, environmental factors—diet in particular—are anticipated to contribute to a greater extent to variations than genetics, although the genetic component may affect the formation of gut profiles during initial stages [33]. Indeed, a study assessing the gut microbiomes of 1046 healthy people with varied genetic backgrounds who populated the same environment revealed that host genetics did not influence the gut microbiota makeup [34]. Pets may influence the gut microbiota by increasing daily exposure to environmental and animal-associated microorganisms through direct contact and shared living spaces. This exposure is associated with greater microbial diversity and shifts in specific gut taxa, particularly during early life, thereby shaping gut microbiota composition. The bacterial diversity of fecal specimens from 332 adult participants living in households with or without pets did not differ; however, notable variations were observed in the prevalence of some taxa, primarily Firmicutes [35]. Exposure to furry dogs changes the gut microbiota profiles of newborns, including an increase in *Oscillospira* and *Ruminococcus*, which have been linked to obesity and an increased risk of developing allergies [36]. Environmental contaminants and food additives are a few examples of chemicals to which humans are regularly exposed; along with substance abuse, these ought to be considered potential moderators of gut microbiota structure. Such foreign compounds that humans may or may not absorb are collectively referred to as “xenobiotics;” once inside the intestines, they affect the growth and functions of gut bacteria. Additionally, some bacteria can metabolize them, producing compounds that differ from the original in terms of function, toxicity, and tenacity [37]. However, this relationship is complicated and still unclear [38]. Physical exercise independently changes the gut microbiota’s constitution and operation; it has been linked to temporary and reversible alterations in the human gut microbiome as well as enhanced butyrate synthesis in animals [39]. For example, the gut microbiome profiles of athletes varied from those of sedentary controls in terms of composition and prevalence, with elevated proportions of Firmicutes and fewer Bacteroidetes [40]. Communication mediated by the vagus nerve, neuroendocrine signaling through the hypothalamic–pituitary–adrenal axis, and serotonin regulation may be the mechanisms by which exercise interacts with gut microbiota [41].

The relationship between metabolic disorders and gut dysbiosis has drawn a lot of attention in the past decade. Whether dysbiosis is the main cause of metabolic dysfunction, a downstream effect, or a component of a reciprocal loop is a key issue yet to be resolved. An increasing volume of research suggests a reciprocative link in which metabolic disruptions are preceded and followed by microbial changes, creating a pathophysiological feedback cycle [42]. Animal models devoid of microbes offer fundamental proof of the gut microbiota’s causative influence on metabolism. In particular, despite being fed less, mice bred normally exhibited a ~60% gain in body fat and resistance to insulin compared to their germ-free equivalents, highlighting the functional role of microbiota in energy storage and preservation [43]. Furthermore, when the microbiota of obese or genetically engineered (TLR5-deficient) mice was transplanted into germ-free mice, it caused hyperphagia, hyperlipidemia, and insulin resistance, hallmarks of metabolic syndrome [44]. Metabolic endotoxemia causes dysbiosis, which elevates gut permeability that, in turn, enables the entry of microbial LPS into the bloodstream. This process activates nuclear factor-κB (NF-κB)- and Toll-like receptor 4 (TLR4)-based signaling pathways, which in turn causes low-grade, chronic systemic inflammation and disrupts insulin-based signaling [45]. Moreover, a decline in bacteria that produce SCFAs, such as *Faecalibacterium prausnitzii* and *Roseburia* spp., impairs gut barrier integrity and induces the release of hormones (e.g., GLP-1 and PYY), which exacerbate metabolic disorders [46]. Recent research using rodent and primate models also links microbially produced amines, such as tryptamine and phenethylamine, to the disruption of insulin responsiveness through trace amine-associated receptor 1 (TAAR1)-based signaling [47].

Conversely, gut dysbiosis is also a result of metabolic diseases such as obesity and T2DM. Dietary patterns characteristic of such conditions—heavy in processed carbohydrates and saturated fats—narrowed microbial diversity while favoring the growth of proinflammatory Proteobacteria [48]. In a human cohort, predatory pathobionts rose, whereas SCFA-producing Firmicutes consistently declined in obese and insulin-resistant populations [49]. Additionally, changes in bile acid metabolism and metabolic inflammation could potentially influence the gut environment, altering the makeup and function of the microbial population [50]. Recent research suggests a reciprocating system of cause-and-effect: dysbiosis leads to metabolic dysfunction, reinforcing and magnifying microbial imbalance. A web of immunological reactions, endocrine routes, and microbial metabolites governs this feedback. For example, research employing machine learning and multiomics platforms demonstrated that particular microbial profiles can forecast the development or course of MetS, providing opportunities for early diagnosis and targeted therapy [51].

Numerous disciplines, including neurology and gastroenterology, have developed disease-specific dysbiosis metrics employing microbial composition patterns to forecast health impacts and propose measures for harmonizing and assessing gut microbial disequilibrium. These indices mark an analytical shift from a binary “dysbiotic against eubiotic” categorization to persistent, clinically meaningful assessments. An enduring inquiry concerning the microbiome is whether gut dysbiosis is an independent contributor to disease pathogenesis or merely a consequence stemming from the associated pathophysiological alterations. Recent developments in quantifiable dysbiosis indices provide a deeper understanding of these concerns, as such scales provide a measure of microbial imbalance and its function.

### 2.1. Stroke Dysbiosis Index

The Stroke Dysbiosis Index (SDI) was constructed to assess changes in the gut microbiota profile in acute ischemic stroke patients. It comprises 18 microbial genera whose relative abundances differentiate stroke patients from healthy subjects. In independent cohorts, SDI exhibited strong discriminatory capacity and was markedly associated with the severity of stroke, early neurological deterioration, and clinical prognosis. In experimental animals, the transplantation of fecal microbiota from high-SDI subjects aggravated inflammation and damage to the brain, microbial dysbiosis, and consequences downstream of the disease process, supporting the notion that it is a primary contributing factor rather than a secondary phenomenon [52].

### 2.2. DiMDI: Microbial Dysbiosis Index in Colitis Models

Dysbiosis severity was quantified under controlled experimental settings using the Dextran Sulfate Sodium (DSS)-induced Microbial Dysbiosis Index (DiMDI). It was originally constructed from a meta-analysis of independent studies involving 11 DSS-induced colitis murine models and comprising 189 datasets, using advanced feature-selection algorithms. It described and characterized Muribaculaceae, and three key genera—*Alistipes*, *Turicibacter*, and *Bacteroides*—for the first time. Independently validated datasets confirmed a predictive accuracy and reproducibility of 82% and 88.9% for DiMDI respectively, validating its robustness as a quantitative dysbiosis measure among various inflammatory disease models. Although DiMDI mainly reflects inflammation-induced microbial alterations, its high reproducibility and strong validation across a multitude of studies provide evidence that dysbiosis has a consistent and numerically persistent pattern that influences the occurrence of these diseases and/or their progression [53].

## 3. Gut Microbiota as Metabolic Gatekeeper

### 3.1. Microbial Metabolites and Host Metabolic Regulation

**Figure 2:**
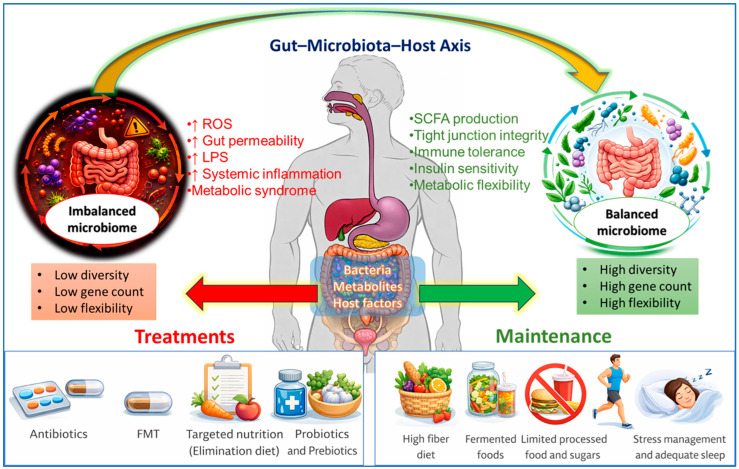
The Gut–Immune–Metabolic axis linking microbial dysbiosis to systemic inflammatory and metabolic disorders. Icons and schematic elements used in this illustration were created using AI-assisted graphic tools and were further modified and assembled by the authors. Red arrows indicate dysbiosis-associated effects, while green arrows represent beneficial microbiome functions; upward arrows (↑) denote increased levels.

Metabolites produced by the gut microbiota can affect its structure and role, as well as host function, either individually or collectively, in a variety of ways. For instance, high concentrations of SCFAs, particularly acetate and butyrate, can suppress the growth of potentially pathogenic and opportunistic bacteria, such as Enterobacteriaceae, *E. coli*, and other facultative anaerobic Proteobacteria, while simultaneously serving as an energy source for beneficial anaerobic commensals, including *Faecalibacterium prausnitzii* and *Roseburia* spp. [54]. These metabolites can also directly alter host functioning by acting on targets that are close to or far from the gastrointestinal system. SCFAs are divided into three categories: butyrate, propionate, and acetic acid (AA). The most common SCFA synthesized by intestinal microorganisms is AA [55], primarily by bacteria, such as *Lactobacillus lactis* and *Bifidobacterium bifidum* [56]. AA preserves the internal environment equilibrium of the gut and aids in maintaining the acid–base balance [57]. Another SCFA, propionate, a product of colonic bacteria fermentation, has anti-inflammatory, anticancer, cholesterol-lowering, and fat accumulation-reducing actions [58]. Butyrate is a vital source of energy in the colon; it is primarily produced by anaerobic bacteria such as *Faecalibacterium* and *Eubacterium rectale* [59], which comprise ~10% of the healthy gut. Additionally, it remarkably reduces inflammation [60]. SCFAs play a crucial role in regulating metabolic disorders, preserving the natural equilibrium of gut microorganisms, and enhancing the host immune system [61]. GPCR41 and 43 are expressed in immune cells, and butyrate activates many G protein-coupled receptors that impact vital metabolic processes [62]. Additionally, they play a crucial role in preserving intestinal health and averting associated illnesses [63]. Figure 2 shows the gut microbiome under healthy conditions and its progression toward dysbiosis-associated metabolic dysfunction, characterized and driven by stress, inflammation, and epigenetic alterations. It also illustrates microbiota-targeted interventions, such as dietary modulation, prebiotics, probiotics, postbiotics, and fecal microbiota transplant, as methods for restoring microbial and metabolic homeostasis.

### 3.2. Amino Acid Metabolism and Metabolic Dysfunction

By producing enzymes that catalyze the synthesis of bioactive metabolites, the gut microbiota controls tryptophan metabolism [64]. When normative and germ-free mice were compared, the former had higher tryptophan availability and lower metabolites of the kynurenine and serotonin pathways, suggesting that tryptophan is catabolized by microbes [5]. The gut microbiota can also convert tryptophan into indole and its byproducts, including indole-3-propionic acid, indole-3-acetic acid, and indole-3-aldehyde (IAld) [65], during which tryptophanase, present in many Gram-positive and Gram-negative bacterial species, such as *Escherichia coli*, *Clostridium* sp., and *Bacteroides* sp., is activated [66]. Furthermore, certain *Lactobacilli* and *Bifidobacteria* can use aromatic-amino-acid aminotransferase- and indole-lactic acid dehydrogenase-dependent routes to convert tryptophan into the intermediate indole-3-lactic acid (ILA) [67]. Branched-chain amino acids (BCAAs) are among the recently discovered compounds produced by microbiota that have lately attracted a lot of scientific interest. In fact, findings linking high blood levels of BCAAs to insulin resistance-related conditions, including diabetes and obesity, and they may even predict the onset of cancer [68]. BCAAs function as either (i) direct building blocks or N donors for proteosynthesis, (ii) an energy/anaplerosis substrate that is eventually broken down to the final glucogenic—propionyl and succinyl-CoA—and ketogenic—acetyl-CoA and acetoacetate—products and oxidized, or (iii) nutritional signals through mTOR activation. Compared to those of hosts, gut bacterial proteins have a larger proportion of BCAAs than other AAs [69]. Critically, the metabolic relevance of these pathways lies not in their individual functions but in their combined ability to influence host inflammation, insulin sensitivity, and epigenetic regulation under dysbiosis.

Higher circulating BCAA concentrations are linked to T2D, obesity, and insulin resistance. A groundbreaking study of 277 insulin-resistant, non-diabetic Danish subjects found that a higher number of genes encoding enzymes that regulate BCAA synthesis was positively associated with insulin resistance; in contrast, genes encoding bacterial influx carriers for these AAs were negatively related. Additionally, it determined that *Bacteroides vulgatus* and *Prevotella copri* were the primary species responsible for an increase in BCAA production. In contrast, *Butyrivibrio crosstus* and *Eubacterium siraeum* decreased the inward transport of BCAAs. Additionally, glutamate—the most effective indicator of elevated body mass index (BMI)—is a consequence of the initial step of BCAA oxidation, in which the amino group is converted to α-ketoglutarate. Finally, four genera of gut bacteria were linked to metabolites that predicted an increased BMI [70]. Leucine, a BCAA, interacts with the mTOR complex, a genetically stable nutrient-sensing system that guarantees optimal metabolic adaptability to energy levels and nutrient availability. Although BCAAs are crucial for suppressing insulin signaling, their overabundance is linked to T2D and insulin resistance; as they are essential AAs and cannot be produced by mammals, this taxon relies on external sources. The gut flora appears to be a significant source of these nutrients in addition to diet [71].

### 3.3. Microbiota–Epigenetic Interactions During Metabolic Disease

Mammalian cells can control gene transcription programs without changing the actual sequence thanks to epigenetics, which refers to heritable variations in gene expression. When determining the genetic basis of illnesses, epigenetic alterations—which mainly include DNA modifications, non-coding RNAs (ncRNAs), and histone modifications—are essential in clarifying the environment–disease interaction [72]. In particular, when combined with genetic information, specific epigenetic markers can indicate proximity to gut microbiota, assisting in the detection of risk for complex disorders [73]. By modulating epigenetic processes such as DNA methylation and histone modification, the gut microbiota can substantially impact host health. Epigenetic modifications can modify gene expression, affecting metabolic and inflammatory processes. Consequently, the gut microbiota–epigenetic interaction is widely acknowledged as a critical element in the emergence of numerous pathologies. Nevertheless, comprehensive convergence on this topic is lacking, and little has been learned about the precise pathways and the degree to which microbiome-induced epigenetic dysregulation is a factor. The gut microbiota modifies DNA methylation to affect host physiology. For example, >200 sites displaying DNA alterations were recognized when juvenile enterocytes were exposed to *Lactobacillus acidophilus* and *Bifidobacterium* [74]. In contrast to GF mice, the IECs of conventionally reared mice exhibited noticeably lower global methylation rates. Furthermore, ten-eleven translocation 2/3 (TET2/3), crucial epigenetic regulatory elements, are hypomethylated when exposed to commensal microbiota, which affects gene expression patterns [75].

Gut bacteria control host DNA methylation via two main mechanisms. First, microbial metabolites, including folate, S-adenosylmethionine (SAM), and SCFAs—such as butyrate and propionate—can either directly donate methyl groups or alter the activity of methylation-involved enzymes [76]. In one instance, folate and SAM produced by *Bifidobacterium* and *Lactobacillus*, respectively, via one-carbon metabolism, increased DNA methylation [77]. Such metabolism depends on -CH_3_ donors like folate, SAM, and methionine (MET); -CH_3_ group transfer is facilitated by key enzymes or coenzymes, such as vitamins B2, B6, and B12 [78]. Second, DNMTs and TETs are directly regulated by microbiota or their metabolites. For instance, the gut microbiota controls TET1 expression to modulate DNA hydroxymethylation; this pattern modifies the epigenetic program of innate lymphoid cell development, impacting intestinal homeostasis and ILC1 expansion [79]. The gut microbiome also influences the post-translational modifications of histone proteins; substantial variations, especially of histone H3 lysine 4 trimethylation (H3K4me3) and H3K27me3, have been observed in GF and antibiotic-treated mice [80]. Additionally, histone H4 acetylation is reduced in the epithelial cells of GF mice [81]. Butyrate, produced by *Clostridia*, works as a powerful intrinsic histone deacetylase inhibitor (HDACi). For instance, microbiota-derived butyrate reduces tuft cell enlargement by modulating histone deacetylase 3 (HDAC3), which is vital for tuft cell hyperplasia, type 2 immunity, and intestinal differentiation [82]. Similarly, independent of the G-protein-coupled receptor system, butyrate promotes B10 cell development by suppressing HDAC and stimulating p38 MAPK, thereby alleviating colitis and arthritis [83]. Major players in the initiation and maintenance of epigenetic changes are non-covalent modifications, especially those facilitated by ncRNAs, such as long non-coding RNAs (lncRNAs) and microRNAs (miRNAs). These ncRNAs are functionally critical modifiers of gene expression in a variety of illnesses and are essential moderators of the epigenetic framework [84]. When GF mice are colonized with microbiota from their pathogen-free counterparts, the miRNA expression profiles of the ileum and colon are disordered [85]. While many mechanistic insights are derived from germ-free or antibiotic-treated animal models, caution is required when extrapolating these findings to humans, given inter-species differences in microbiota composition and metabolic regulation.

### 3.4. Circadian Rhythms, Environmental Factors, and Microbial Oscillations

According to findings from investigations on humans and animals, host circadian rhythms significantly impact oscillations in microbial distributions and functions, despite not being exposed to light [86]. Unfortunately, little is known about the intricate relationships between microorganisms and host variables that control circadian rhythm oscillations. The core molecular clock machinery can markedly impact the signals of both central and peripheral metabolic regulation. Remarkably, during a typical light–dark cycle, microbial α-diversity in *Per2* knockout (KO) mice is remarkably greater than that in wild-type (WT) mice. Additionally, Lachnospiraceae and Ruminococcaceae are more prevalent when *Per2* is knocked down, but *Erysipelatoclostridium* and *Olsenella* spp. are less prevalent [87]. Furthermore, animals lacking *Per1/2* show virtually no rhythmic variations in commensal bacterial abundances, including Bacteroidales. Furthermore, *Per1/2*-deficient mice lose diurnal patterns in metagenomic pathways—such as cell wall production and vitamin and nucleotide metabolism—normally seen in WT mice. Compared to WT mice, *Clock*-KO animals had a higher proportion of Firmicutes/Bacteroidetes as a result of this modification [88].

External variables, such as environmental signals and stimuli, significantly impact the circadian rhythms of gut microorganisms by influencing not only the timing but also the extent of microbial activity. Light is a crucial external factor influencing chronological rhythm because it helps many organisms, including microbes, synchronize their internal clocks. Microbial metabolic activities and gene expression trends change in response to the triggering of particular biochemical cascades by the presence or absence of light. Moreover, dietary habits also contribute to aberrant diurnal variations and imbalance. The fluctuations in the composition, function, and rhythmicity of the gut microbiota are impacted by a variety of factors, including routine snacking, time-restricted food intake, late-night eating, abrupt shifts in the diet’s fat and fiber composition, and intake of food with fiber and other unprocessed components [89].

## 4. Microbial Dysbiosis in Specific Metabolic Disorders

### 4.1. Gut Dysbiosis and Obesity

The incidence of obesity is steadily increasing worldwide, making it a global health issue. Higher cardiometabolic risks, such as T2D and moderate inflammation of enlarged fat deposits, are linked to obesity. Obesity is defined by an imbalance in energy induced by several factors, including a vital instability in appetite and satiation, as well as numerous biological, histological, immunological, and metabolic alterations in adipose, hepatic, muscular, brain, and intestinal tissues [90]. Over the past decade, efforts have focused on clarifying microbial signaling networks that may affect host physiology and either cause or sustain obesity. However, early data indicated an enhanced likelihood of gut dysbiosis in obese patients. Gut bacteria control a wide variety of physiological and pathological processes, including obesity, homeostasis, inflammation, and insulin resistance by synthesizing different metabolites, compounds, proteins, and peptides. Therefore, any change from the wholesome cohabitation of microbiota–host to a chronic imbalance may be caused by excessive fat deposition and the subsequent difficulties. Alterations in the gut microbiota diversity may remarkably influence the underlying causes of obesity. In a leptin-deficient (ob^−^/ob^−^) obese mouse model, 16S rRNA gene sequencing identified an enormous spike in Firmicutes and a decrease in Bacteroidetes [91]. Accordingly, further investigation on obesity has linked it to a decline in *Bifidobacteria* and a rise in certain bacterial species, like *Halomonas* or *Sphingomonas* [92]. Lower bacterial gene counts are linked to high adiposity, insulin resistance, and dyslipidemia, which are characteristics of obese patients, and the diversity of gut microbiota varies in healthy people. Obese people have a greater proportion of Firmicutes and a lower percentage of Bacteroidetes [93]. In a study, people with a Firmicutes/Bacteroidetes proportion ≥ 1 were 23% more likely to be overweight than those with a proportion < 1 [94]. The primary drawback of the F/B ratio is its oversimplification of microbial ecology. The phyla Firmicutes and Bacteroidetes have countless functionally diverse species with varying metabolic capacities. Significant strain-level variations and ecological relations, more closely linked to host physiology, are obscured when these varied species are grouped into two broader groups. Numerous studies have explored the F/B ratio as a reliable signature of gut microbiota dysbiosis during metabolic diseases such as T2DM and obesity. However, notable discrepancies across demographics, analytical techniques, and medical situations severely restrict its usefulness as a ubiquitous predictive or prophetic metric. Recent studies suggest that the F/B ratio’s significant inter-individual and inter-population volatility renders it insufficiently robust for therapeutic use [95].

### 4.2. Metabolic Endotoxemia and Inflammatory Signaling

Initially, Gram-negative bacteria generate LPS that can penetrate the intestinal epithelium via chylomicrons or broken TJs [45]. Obesity and an HFD alter gut microbial diversity and the configurations of TJ proteins, facilitating LPS passage. The CD14 receptor is activated when LPS binds with the LPS-binding protein (LBP) in the circulatory system. When juxtaposed with saline-infused animals, LPS infusion replicates the characteristics of HFD-fed mice in terms of body weight, weight gain, abdominal and subcutaneous adiposity, elevated fasting blood sugar levels, insulinemia, and hepatic triglyceride content [96].

### 4.3. Gut Microbiota Alterations in T2DM

Recent investigations have offered fresh insights into how microbial dysbiosis contributes to the pathophysiology of T2DM [97]. While variances in gut microbial β-diversity are linked to insulin resistance, greater α-diversity in the gut microbiome was associated with reduced insulin resistance and fewer cases of T2DM. Additionally, a rise in the number of 12 specific taxa can mitigate insulin resistance and the likelihood of T2DM through the production of butyrate [98]. People with concomitant sensitivity to glucose might already have altered gut flora, which emphasizes the possibility of microbial signatures for the early detection of people at high risk of developing T2DM [99].

In T2DM, strain-specific gut microbial fingerprints were recognized in 8117 metagenomes from an updated multinational population assessment. The research linked five species to T2DM and 14 to prediabetes and T2DM. Of these, two species—*Coprococcus eutactus* and *Turicibacter sanguinis*—were deficient in T2DM, whereas three species—*Clostridium citroniae*, and *E. coli*—were elevated in T2DM. The abundance of several species rose steadily or fell in T2DM patients, prediabetic people, and normoglycemic controls. Additional retrospective and experimental research is required to corroborate the findings that changes in gut microbiota served as an antecedent of T2DM development [100]. At the genus level, Asian people with T2DM exhibited fewer strains of *Bacteroides*, *Faecalibacterium*, and *Blautia* and higher percentages of *Bifidobacterium*, *Streptococcus*, and *Prevotella* than healthy controls [101]. *Prevotella* levels were likewise enhanced in US T2DM patients. However, they usually exhibited elevated proportions of *Bacteroides*, *Faecalibacterium*, and *Blautia* than Asian patients and healthy persons. Furthermore, *Alistipes* was detected in these patients at a decreased abundance [102]. Notably, not all groups experience the same shifts. Environmental factors, genetic background, and food are examples of host traits that present a significant regulatory impact. People with T2DM additionally demonstrated substantial alterations in microbial functions and in bacterial composition. Patients with T2DM exhibit more opportunistic infections and fewer butyrate-producing bacteria [98]. The gut microbiota patterns of patients with prediabetes and T2DM were included in a population-based study conducted in Sweden. In either the discovery and validation groups, this study continually observed that while the abundance of *Sporosarcina newyorkensis* was higher, the prevalence of *Clostridium thermocellum*, *Peptoniphilus* sp. Pral taxon 375, *Heliobacterium modesticaldum*, *Syntrophobotulus glycolicus*, and *Clostridium* sp. L2-50 was lower in people with T2DM. The majority of these reduced bacterial taxa belong to Firmicutes. Furthermore, according to another study involving the Dutch population, >50% of the metagenomic species altered in patients with T2DM were potential butyrate producers, including *Akkermansia*, *Clostridium*, and *Faecalibacterium*. A greater proportion of 12 butyrate-producing gut bacteria might lower insulin resistance and T2DM incidence. Advancements in multiomics technology have enabled a thorough understanding of the pathophysiology of T2DM. Although metabolomics and proteomics reveal changes in the host’s interaction networks and compounds originating from the microbiota, metagenomics makes it easier to identify certain microbes linked to T2DM [103].

### 4.4. Gut Dysbiosis and Nonalcoholic Fatty Liver Disease (NAFLD)

Numerous studies have demonstrated the role of several gut microbiota pathways in the development of NAFLD [46]. Enzymes produced by the gut microbiota are essential for the breakdown of dietary fibers into SCFAs and undigested polysaccharides into monosaccharides. This mechanism is critical because it provides the host cells with the necessary metabolic backing [104]. AA provides ~10% of the body’s daily energy requirements, making it an important energy source. In contrast, butyric acid plays a critical role in maintaining intestinal barrier functionality by supplying energy to sustain epithelial cells. Apart from the host genotype and disease-specific characteristics, numerous other factors may influence the rhythmicity and stability of a microbial community. Metabolites from the gut and intestinal microbes are prominent dietary factors influencing intestinal microbial composition, particularly when the levels of intestinal microbes and products of microbial biosynthesis are altered. Among others, disturbed sleep, shift work, and irregular eating cause circadian rhythm disorders that result in the non-synchronization of microbial rhythms [105]. In particular, this manifests as a decline in certain metabolic pathways, and an increase in oxidative stress and inflammation [105,106]. Moreover, antibiotic use, stress, psychosocial stress, lack of physical exercise, environmental pollutants, cultural variations, and diets from different geographical areas create a broad diversity in gut microbiomes across populations globally [107]. Additionally, it provides 60–70% of the energy needed for development and proliferation, serving as a principal metabolic substrate for the gastrointestinal microbiota [108]. Furthermore, butyric acid can reverse the stimulation of sterol regulatory element binding protein-1 (SREBP-1) and carbohydrate response element binding protein (ChREBP), in turn inhibiting lipogenesis [109]. Propionic acid is usually catabolized in the liver, where pyruvate is metabolized into glucose. It reduces hypercholesterolemia in overweight or obese people. It is an initiating agent for adipogenesis and gluconeogenesis, in contrast to butyric and acetic acids, that deliver energy to host cells [110]. The progression of NAFLD is more significantly influenced by subsequent processes [111]. Mitigation of gut dysbiosis relates to the advancement of NAFLD through the microbiome’s impact on gut barrier function, metabolites, and chronic liver inflammation. Dysbiosis of the gut microbiome is thought to reduce butyrate production and weaken the gut junctions. Increased gut permeability and translocation of microbes via portal circulation to the liver enhance its exposure to LPSs. This, in turn, causes the liver to produce inflammatory signals, increasing oxidative stress, insulin resistance, and lipid accumulation. In addition, dysbiosis modifies SCFAs levels and bile acid metabolism, impairing FXR and TGR5 signaling. These changes disrupt the homeostasis of lipids and glucose. Lastly, the enhanced microbiome synthesis of trimethylamine (TMA) and its oxidation to trimethylamine N-oxide (TMAO) by the liver ties gut dysbiosis to endothelial dysfunction and cardiometabolic risk factors associated with NAFLD, confirming dysbiosis as an impact disease catalyst.

Clinical trials have shown that a reduction in choline induces fat deposition in the liver. A decrease in the hepatocyte ability to produce and disperse very low-density lipoproteins (VLDLs) causes steatohepatitis. This result is frequent when rodents are fed a methionine and choline-lacking diet (MCD). TMA lytic enzymes in bacteria belonging to Proteobacteria and Firmicutes degrade dietary methylamine, choline, phosphatidylcholine, and carnitine into a variety of metabolites, including TMA. TMA is delivered to the liver through the portal vein, where it is transformed into TMAO by flavin-containing monooxygenases. TMAO is essential for promoting the accumulation of activated leukocytes in human endothelial cells. Thus, this process compromises endothelial cell function, significantly increasing vulnerability to atherosclerosis and cardiovascular disorders [112].

### 4.5. Gut Microbiota and Polycystic Ovarian Syndrome (PCOS)

Though the pathophysiology of PCOS remains unknown, numerous investigations have demonstrated that the gut microbiota is crucial to its onset and development [113]. In rats with dehydroepiandrosterone/high-fat diet-induced PCOS, gut Bacteroides were reduced while Firmicutes and *Proteus* elevated. Correlation modeling revealed an association between the quantities of gut microbiota and inflammatory factors in mice [114]. Letrozole-induced PCOS rats had more pullorum and fewer intestinal *Lactobacillus*, *Ruminococcus*, and *Clostridium* than the healthy control group [47]. The gut microbial diversity, composition, and intestinal mucosal barrier were altered in patients with PCOS [115]. Obese PCOS patients had elevated levels of Enterobacteriaceae and lower contents of *Lactobacillus* and *Bifidobacteria*; alterations in gut microbiota were linked to enhanced insulin resistance and inflammatory levels when compared to non-obese PCOS patients and the healthy control group [116]. Patients with PCOS with or without insulin resistance have different gut microbiota compositions and structures [117]. These investigations demonstrated that patients with PCOS have altered gut microbiome diversity and quantity of associated bacteria. By contributing to an inflammatory response in patients with PCOS, the gut microbiota could affect the intestinal mucosa’s integrity and subsequently impact its metabolism [117]. Abnormal sex hormone levels, insulin resistance, polycystic ovarian alterations, persistent subclinical inflammation, and other traits are typical of PCOS [118]. Endotoxemia, SCFA synthesis, bile acid metabolism, aberrant release of brain–gut peptides, and other conditions are impacted by gut microbiota dysfunction.

Hyperandrogenism, resistance to insulin, persistent inflammatory responses, and aberrant levels of brain–gut peptides are among the physiological and pathological processes linked to PCOS symptoms mentioned above [119]. The pathogenic etiology of PCOS includes the brain–gut axis in addition to the hypothalamus–pituitary–ovary (HPO) axis failure [120]. A biphasic neural signaling system constitutes the brain–gut axis. It establishes a robust connection between the gut and the brain. It is an essential component of the information transfer network [121]. Gut microbiota disorders may contribute to PCOS development through this axis [122]. SCFA, which intestinal bacteria can produce, helps intestinal endocrine cells secrete brain–gut peptides, such as glucagon-like peptide 1, growth hormone-releasing peptide (Ghrelin), and YY peptide. Using G-protein coupled receptor 43, SCFA promotes transcriptional activation of signal pathways, mammalian rapamycin targets protein/signal transduction, and controls the metabolism of such peptides [54]. Ghrelin and other peptides regulate the release of luteinizing hormone and the hypothalamic regulatory nucleus. By stalling the pituitary’s luteinizing hormone-releasing pulse exertion, ghrelin can also prevent the overproduction and release of luteinizing hormone, which helps control the reproductive system-related function of PCOS [123].

### 4.6. MetS, Age, and Geographic Variability

Given that the microbiota is closely linked to the development of significant illnesses like type-2 diabetes mellitus (T2DM) and cardiovascular diseases (CVD), metabolic syndrome (MetS) has gained global interest. Additionally, its incidence in children and adolescents has been rising alarmingly in recent years [124]. Early identification and treatment are essential to prevent the development of diseases and major health issues in the future because MetS is treatable and even preventable. Considering that findings in mice indicate a possible connection between gut microbiota and MetS, microbiome-based therapies have gained favor recently as a means of treating and preventing metabolic disorders [44]. Additionally, studies in humans have uncovered novel perspectives on gut microbiota–host interactions; however, the majority have focused on adults owing to ethical and practical constraints or even challenges in collecting samples from children. Regardless of children’s gut microbiota being more adaptable to changes induced by environmental factors (such as diet) than adults, irrespective of the opinion that the consolidation of gut microbial diversity occurred at age 3 [125]. On average, MetS comprises three categories of cardiometabolic abnormalities, also referred to as “Syndrome X,” an acquired condition: higher central adiposity, hyperglycemia, elevated triglyceride levels, and reduced high-density lipoprotein cholesterol (HDL-c) [126]. The association between gut microbiota and MetS was noteworthy a decade ago. *16S rRNA* sequencing revealed that Toll-like receptor 5 KO mice (T5KO) exhibited gut microbial changes associated with elevated low-grade proinflammatory signaling, resulting in MetS. This association was confirmed by transplanting gut microbiota from T5KO into WT germ-free mice, in which all essential characteristics of MetS appeared soon after treatment [44]. Differences in the maturation of human gut microbes start at birth. The maternal gut microbiota is crucial for a newborn, as evidenced by the apparent effects of baby feeding and delivery mode on gut colonization [127]. Numerous studies have already defined the microbiota of the human gut in adults and in children aged 0–3, but none in those aged 3–18 because of sample collection challenges or practical or ethical concerns.

The gut microbiota of school-age children and their relationships to MetS and T2DM are the subject of little or no research; in contrast, obesity and overweight are among the most addressed metabolic disorders in this demographic [125]. In contrast to investigations that found a higher abundance of Firmicutes and a lower percentage of Bacteroidetes in healthy American children aged 7–12, the former had an elevated number of *Bifidobacterium* spp. and members of the Bacteroidetes phylum—which includes the genera *Bacteroides* and *Prevotella*—whereas the latter had a greater abundance of the Firmicutes phylum, such as the genera *Eubacterium*, *Clostridium*, *Dorea*, and *Coprococcus*. These findings suggest that geographical distribution may also affect gut microbiota–host interactions [128], through diet and lifestyle, environmental exposure, and early-life practices that modify microbial composition and function. For example, people consuming traditional diets rich in fiber have different microbial taxa and metabolic activities that vary in SCFAs, bile acid metabolism, and energy homeostasis, in contrast to people consuming a high-fat and high-sugar Western diet. Other geographically distributed factors, such as sanitation, climate, urbanization, antibiotic use, environmental microbial exposure, and infant exposure (or lack of it) to microbes, can affect early colonization and the eventual stability of the microbiome. The genetic background of the host, cultural practices, and even infant feeding styles also affect microbiota composition. All factors mentioned above can influence the metabolic and immune functions of the microbiome, as well as pathogenicity.

Furthermore, the gut microbiota of children is more adaptable to environmental changes and is rich in functions that could aid in the growth of the host, whereas the gut microbiota of adults is more susceptible and has more inflammatory, adipose, and obesity functions [129]. Since the gut–immune system activates pattern recognition receptors (PRRs) in response to environmental changes and endogenous threats, pathogen-associated molecular patterns (PAMPs) induce reactions. The family of PRRs known as toll-like receptors (TRLs) can sense PAMPs and a wide range of microbial- and damage-associated molecular patterns (MAMPs and DAMPs, respectively). Due to their presence in the membranes of immunological and gut epithelial cells, TLR2, TLR4, and TLR5 interact the most with the human gut microbiota [130]. Table 1 provides an overview of the microbiome bacteria principally linked to disorders and the methods employed to study them, including *16S rRNA* gene sequencing, shotgun metagenomics, quantitative PCR, and culture methods. The table also indicates differences in study design—human observational, interventional, and animal—which may explain some of the heterogeneity in the reported microbial associations.

## 5. Gut–Immune Metabolic Axis

**Figure 3:**
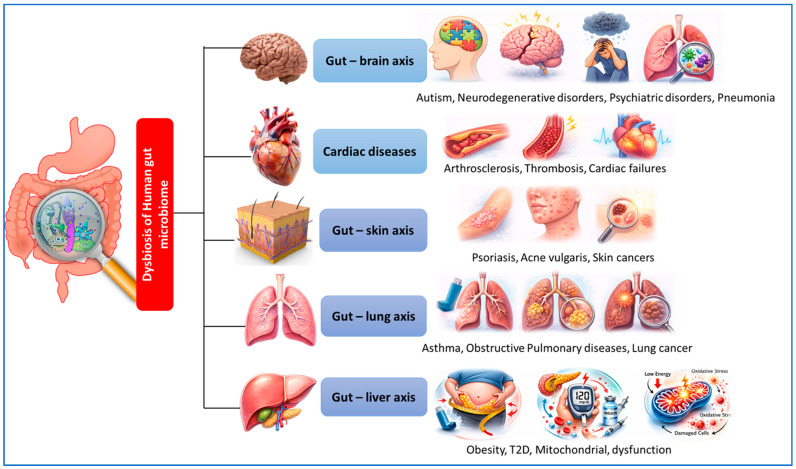
Conceptual overview of gut microbiome homeostasis and progression toward dysbiosis-associated metabolic dysfunction. Icons and schematic elements used in this illustration were created using AI-assisted graphic tools and were further modified and assembled by the authors.

A rapidly evolving field of study is the association between the host immune system and gut microbiota, or bacteria that reside in the human gastrointestinal tract (Figure 3). Gut microbiota-derived metabolites function in inflammatory signaling and communicate with host immune cells, directly or indirectly. The gut microbiota has a crucial role in controlling inflammation and immunity within the host. Dietary probiotics boost immunity and diminish inflammatory responses in host animals [139].

### 5.1. Gut Microbiota as an Architect of Intestinal Immunity

Gut-associated lymphoid tissue (GALT) and diffuse immune cells are crucial components of the immunological barrier. GALT can suppress aberrant immune responses and maintain host immunity by identifying and eliminating pathogenic bacteria. The establishment of immune tolerance involves TLRs and the nuclear factor kappa-B (NF-kB) signaling pathway [140]. Commensal bacteria-derived metabolites profoundly affect immune cells, such as dendritic cells (innate immunity) and classical T lymphocytes (adaptive response). T cells’ localized involvement aids the immune system in launching prompt effector responses. Complex, dynamic, and context-dependent connections exist between host immunity and gut microbiota [141]. The host microbiome specifically develops inherent and adaptive immune systems, which orchestrate the maintenance of elements critical to host–microbe synergy [142]. The intestinal immune system is primarily composed of native and adaptive immune cells, specialized epithelial cells, mesenteric lymph nodes, gut bacteria, and associated metabolites [143]. GALTs are parts of the mucosa-associated lymphoid tissues (MALTs), which are a direct conduit between the host and the environment. The gut mucosa’s initial line of defense is provided by the innate immune cells found in GALTs. The primary histological elements of GALTs are the appendix, isolated lymphoid follicles, Peyer’s patches, crypt patches, and mesenteric lymph nodes [144]. The gut microbiota alters the morphological growth of GALTs and triggers their immune activity to initiate host defense mechanisms; it sustains tolerance toward commensal bacteria via Pattern Recognition Receptors (PRRs)–PAMP recognition and epigenetic modulators like SCFAs. GALTs, especially mesenteric lymph nodes, are the first to initiate gut-driven immune responses characteristic of the pathogenesis of autoimmune diseases, potentially altering the immune response at the organizational level [145].

As the main contributors to cell-based immunity, adaptive T cells maintain the host’s homeostasis against the disruption caused by inflammatory disorders induced by the immune system. The gut microbiota can stimulate T-cell growth, causing them to generate adaptive immune responses and respond swiftly to cues from the intestinal lumen environment. The commensal microbiota influences T cell development, function, and differentiation, thereby preserving host immunological homeostasis [146]. The key role of T cells—which are further subdivided into CD4^+^ and CD8^+^ T cells—is controlling adaptive immune responses. A specific commensal microbiota induces Th cell polarization and cytotoxic CD8^+^ T-cell activity [147]. However, a specific strain of *Fusobacterium varium* markedly alters the intestinal adaptive immune phenotype upon monoclonal colonization. Compared to other bacteria, *F. varium* significantly reduced the number of CD4^+^ and CD8^+^ T lymphocytes and increased the proportion of colonic double-negative cells (CD4^−^ CD8^−^ TCRb^+^). Additionally, a wide range of genes involved in bile acid metabolism—which are closely related to immune function—were markedly suppressed by *F. varium* [148]. The microbial environment serves as a T cell antigen, and some microbes, including segmental filamentous bacteria, help produce thymic T cells that are unique to the gut microbiota. The main function of these cells is to control adaptive immune responses [149]. CD8^+^ T cells are essential for tumor monitoring and defense against intracellular invaders such as bacteria and viruses [150]. Microbial metabolites modulate CD8^+^ T-cell function; butyrate and propionate, two significant SCFAs, control the production of IL-12 by antigen-presenting cells (APCs), thus limiting the proliferation of CD8^+^ T lymphocytes [151].

### 5.2. Innate Immune Signaling and Microbial Pattern Recognition

*Megasphaera massiliensis* produces pentanoate, which enhances effector CD8^+^ T-cell activity; higher production of TNFα and IFNγ indicated that *M. massiliensis* increased the effectiveness of adoptive T-cell therapy [152]. The use of GF animal models provides valuable insights into the causal molecular links between commensal microbiota and host immunity. Early research on GF mice showed that significant abnormalities of intestinal lymphoid tissue architecture and immunological activities are linked to a lack of commensal bacteria [153]. In comparison to traditional colonized animals, GF mice exhibit a considerable decline in αβ and γδ intraepithelial lymphocytes (IELs), which can be markedly increased following *de novo* colonization [154]. A key component of protective humoral mucosal immunity, IgA antibodies, decline remarkably in GF animals and neonates but are quickly recovered upon microbial colonization [155].

Prominent groups, including *Salmonella typhimurium*, *Helicobacter pylori*, *Porphyromonas gingivalis*, *Escherichia coli*, and *Klebsiella pneumoniae* are examples of Gram-negative bacteria found in the environment. LPS, commonly referred to as endotoxin, is a complex glycolipid consisting of a hydrophilic polysaccharide and a hydrophobic lipid A moiety. It is an essential pathogenic component of the cell wall [156]. It functions as a key antigen PAMP throughout the early stages of infection, affecting the transcription of several macrophage genes and inducing a range of immune responses that lead to systemic inflammation [157]. TLR4 recognizes LPS and sets off a complicated series of immunological reactions. There LPS–TLR4 intracellular signaling chain functions via two main pathways. MyD88, TIR domain-containing adapter protein (TIRAP), interleukin-1 receptor-associated kinases (IRAKs), TNF receptor-associated factor 6 (TRAF6), TAK-binding proteins (TABs), and TGF-β-activated kinase 1 (TAK1) are all sequentially engaged in the MyD88-dependent pathway, which culminates in the activation of NF-κB and activator protein-1 (AP-1). In contrast, the MyD88-independent pathway uses TRIF-related adapter molecule (TRAM), TIR domain-containing adapter molecule 1 (TICAM1, or TRIF), TRAF3, TRAF family member-associated NF-κB activator, inhibitor of nuclear factor kappa-B kinase subunits γ/ε, and TANK-binding kinase 1 (TBK1) to activate NF-κB and interferon regulatory factors, and finally type-I interferon responses [158].

### 5.3. Immune–Metabolic Crosstalk and Metaflammation

Westernized lifestyle, which is defined by inactivity and an energy-dense Western diet high in fat and simple sugars, is linked to “metaflammation,” a persistent metabolic inflammation that is particularly noticeable in obese people regardless of any infection [159]. Nevertheless, the precise causes, its fundamental molecular processes, and pertinent cell signaling pathways are still scattered and unknown. Significantly, metabolic diseases cause havoc in the body’s internal environment and are associated with metabolite surpluses and cell death or injury, which are partially caused by disruptions in the gut microbiota [65]. Disturbances in glucometabolic pathways, prevalent in individuals with obesity and T2DM, have been widely associated with low-grade inflammation. Subjects with MetS have enhanced blood levels of many cytokines, which negatively impact peripheral tissues processes [160]. For instance, compared to those who are insulin-sensitive, individuals who are insulin-resistant and glucose-intolerant had an elevated proinflammatory profile and a different reaction to respiratory viral infections [161]. In a similar vein, the gut microbiome was linked to more severe asthma and a greater inflammatory tone in obese people [162]. The findings on the theory that microorganisms mechanically travel to regions where they could cause an inflammatory reaction are conflicting. The production of gut-derived compounds that enter systemic circulation induce a number of consequences that are a part of an expanded contemporary stream of perception that links gut microbiota to inflammation and cardiometabolic disease. Certain gut bacteria have been connected to these metabolites. A number of these metabolites have drawn a lot of interest in recent years [163]. A direct link between metabolism, the immune system, and gut microbiota has been established only for a few bacterial metabolites; however, compounds such as imidazole propionate have been identified recently and implicated in insulin resistance [164].

## 6. Therapeutic Interventions: From Probiotics to Postbiotics

Probiotics have been studied for centuries. The latest description provided by a collaborative FAO–WHO committee is “living microorganisms which, after administration in sufficient quantities, offer a health benefit.” The term “probiotics” was originally used to refer to microorganisms and molecules that aid in gut microbial equilibrium, and has since undergone multiple modifications. At first, researchers concentrated on using probiotics to treat and prevent gastrointestinal diseases by replacing the specific causative bacterial strains and stopping the proliferation of pathogens [165]. Next-generation probiotics (NGPs) and live biotherapeutic products (LBPs) with superior characteristics, like those generated through synthetic biology, have emerged recently [166]. According to comparative microbiota assessments, NGPs are live bacteria that, when provided in sufficient quantities, boost the host’s health. However, LBPs are not vaccinations; rather, they are biomaterials that include living organisms and can be used to mitigate, ameliorate, or alleviate human diseases or conditions [167]. They constitute microbial genera and species that were not previously utilized in the food or pharmaceutical industries, and are identified by next-generation sequencing and bioinformatics [26].

### 6.1. Probiotics and Cancer Prevention

The prospect of NGPs for combating numerous cancers, such as colorectal, gastric, and cervical cancer, and hepatocellular carcinoma, has been investigated. However, additional studies are required to determine whether probiotics are effective in addressing certain tumors [168]. Greater vulnerability to tumors can result from changes in the gut microbiota caused by inflammatory and carcinogenic stimuli. Probiotics might function against CRC by promoting barrier function and epithelial repair; interfering with inflammatory pathways involved in tumorigenesis; inducing apoptosis; upregulating the production of cytokines and immunomodulatory metabolites such as SCFAs, acetate, and propionate; inhibiting biofilm formation; and selectively excluding pathogenic and tumor-causing microorganisms [169]. Furthermore, *Lactobacillus reuteri*, a gut bacterium that produces histamine, decreases inflammation and hinders the growth of colon tumors [170]. Additionally, putrefactive carcinogenic enzymes are inhibited by lower intracolonic pH caused by *Lactobacilli* [171]. Epidemiological investigations on *Bifidobacterium infantis*, *Bifidobacterium breve*, *Bifidobacterium longum*, *Streptococcus thermophilus*, *L. acidophilus*, *L. salivarius*, *L. plantarum*, *L. rhamnosus*, *L. kefiri*, *L. casei*, and *L. delbrueckii* demonstrated encouraging outcomes in preventing malignancies and early-stage colon cancer in animal models [169]. *Prevotella copri*, *Christensenella minuta*, *Parabacteroides goldsteinii*, *Akkermansia muciniphila*, *B. thetaiotaomicron*, *F. prausnitzii*, and *B. fragilis* are among potential NGPs identified in recent research that may offer a variety of health advantages [172].

### 6.2. Mechanisms of Probiotic Action in Gastrointestinal Disorders

Although the exact processes by which probiotics work against gastrointestinal illnesses are unknown, several theories have been postulated in light of recent studies. Probiotics compete with detrimental microbes for resources in the gut, minimizing their proliferation and function. This effect enhances gut health and ameliorates the symptoms of a variety of GI disorders. It is one of the most often used pathways for explaining the effect of probiotics in GI ailments. Another approach, especially in IBD and other disorders involving gut inflammation, is the activation of the immune system to lower inflammation [173]. *B. pseudopodium*, *L. rhamnosus*, *L. acidophilus*, and *L. lactobacillus* are among the most widely utilized NGPs; they cure GI issues by modifying the serotonergic system in IBS. In a mouse model of low-grade inflammation and intestinal dysfunction brought on by dinitro-benzene sulfonic acid, *F. prausnitzii* lowered intestinal permeability, colonic serotonin, and cytokine levels [174]. Furthermore, *B. bifidum* lessens the chances of antibiotic-associated diarrhea and alleviates signs of IBS, such as bloating and abdominal pain [175].

### 6.3. NGPs in Cardiometabolic Disorders

Recent research has highlighted the importance of regulating gut microbiota for cardiovascular health, creating opportunities to investigate NGPs as preventive and therapeutic agents. The impact of gut microbiota on inflammatory responses, sensitivity to insulin, and cholesterol levels demonstrates its substantial role in cardiovascular health [176]. NGPs can preserve a healthy gut microbiota through selective stimulation, providing a strong argument for their use in the prevention and therapy of cardiovascular disorders. *P. copri* and *C. minuta* are notable for their ability to control insulin resistance, a major contributive factor to cardiovascular disorders [177]. A wide range of NGPs helpful in cardiovascular health is also influenced by Eggerthellaceae bacteria, such as *Eggerthella lenta*, which converts ellagitannins from particular meals into metabolites with cardioprotective and anticarcinogenic properties [178]. The potential influence of NGPs on cardiovascular health is highlighted by the recognition of *B. thetaiotaomicron*’s significance in correcting insulin resistance and obesity [179]. *B. lactis* is another intriguing NGP useful for treating metabolic illnesses; it enhances lipid metabolism, lowers hemoglobin A1c levels, improves insulin sensitivity, and decreases body weight [180]. *B. animalis* subsp. *lactis* BB-12 improves the human gut flora; this probiotic strain fights obesity and preserves community structure in the gut microbiota. It is accomplished by inhibiting the growth of pathogens, including *Clostridium* sp., *Blautia* sp., and *Bacteroides* sp., and encouraging commensals, such as *Prevotella* sp. Consequently, the shift from a state of health to one of obesity was inhibited [181]. Furthermore, *B. longum* lowers inflammation in patients with metabolic disorders and enhances glucose metabolism and body weight. However, milk fermented with *B. longum* 070103 considerably lowers 3-indoxyl sulfate, an effect detrimental to the intestinal barrier. Additionally, mice treated with BLFM showed improvements in hepatic steatosis, insulin resistance, glucose tolerance, and BW [182]. A connection between higher levels of *B. longum* and *B. bifidum* with fatty liver, body mass index, blood triglycerides, and visceral adipose tissue (VAT) has been proposed. The carbohydrate and nucleoside metabolic processes of these *Bifidobacterium* strains can prevent diet-induced obesity by enhancing bile acid-based signaling, reducing body weight gain while improving hepatic steatosis and glucose regulation. By modifying intestinal sterol biosynthesis mechanisms, these strains also shield germ-free mice from diet-induced obesity [183].

### 6.4. Fecal Microbiota Transplantation as a Therapeutic Strategy

In an unbiased exploratory study involving extensive fecal microbial transplantation (FMT) in obese people, after intensive FMT intervention, nine recipients lost weight to varying degrees at the 12-week follow-up. This observation suggests that the rigorous FMT induced moderate and variable weight loss. FMT can modulate the intestinal mucosal microbiomes of obese patients; the duodenal mucosal microbiome remained similar, but the colonic mucosal microbiome saw more substantial modifications. Weight loss in obese patients following FMT intervention may be linked to recovery of mucosal Bacteroides [184]. Additionally, FMT can alter fat distribution and lower visceral fat. The android—gynoid (A/G) fat ratio decreased slightly following FMT intervention, but the BMI SDS did not alter significantly. This finding suggests that visceral obesity may have declined, thus lowering the abdominal fat percentage, while altering fat distribution [185]. By variably regulating the enteroendocrine axis and restoring physiological routines for GLP-1 secretion via the implantation of specific bacterial taxa, FMT in conjunction with low-fermentable fiber may enhance insulin sensitivity [186]. However, in week 18 following FMT, neither single nor recurrent allogenic FMT significantly influenced susceptibility to peripheral or hepatic insulin, indicating no prolonged effects on insulin resistance. The human immune system adjusting to the altered gut microbiota following FMT could account for this long-term alteration [187].

### 6.5. Postbiotics: Non-Viable Microbial Therapeutics

A substitute biotherapeutic product featuring inanimate (non-living and non-viable) bacteria, cell components derived from microorganisms, and metabolites has garnered attention. These products may be more prudent for use among susceptible groups, such as infants and young children; patients with immunocompromised or critically ill conditions; and those with intestinal barrier dysfunction, with more benefits than probiotics. We refer to these biotherapeutic drugs as postbiotics, a word that has gained the most traction in recent years [188]. While the precise term is controversial, these are characterized as non-living bacteria that continue to perform their primary role and enhance the host’s health [189]. Fermented infant formulas and paraprobiotics, also referred to as “ghost probiotics,” are common forms of postbiotics, also called metabiotics. Usually devoid of live bacteria, such infant formula is a precursor or follow-on formula that was fermented using bacteria or other microorganisms that produce lactic acid [190]. Postbiotics are complex preparations that comprise a variety of bioactive compounds with various modes of action; in most cases, it is unclear how they contribute to human health. Postbiotics are thought to work in five primary ways: (a) altering the composition of resident microbiota; (b) enhancing epithelial barrier function; (c) altering local and systemic immune responses; (d) influencing systemic metabolic responses; and (e) influencing systemic signaling via the nervous system [189]. The immunoregulatory impact of postbiotics derived from *L. rhamnosus* CRL 1505 on human intestinal epithelial and dendritic cells was assessed following LPS stimulation. In DCs, CRL1505 enhanced the synthesis of TNF-α, IL-1β, IL-6, and IL-10 while decreasing the expression of CD40, CD80, and CD86. In human IECs and DCs, the TLR4-triggered immunological response was regulated by peptidoglycan (PG1505) derived from *L. rhamnosus* CRL1505 [191]. As a lipid regulator that can decrease fat via the insulin-like growth factor (IGF-1) pathway, lipoteichoic acid from *Bifidobacterium animalis* subsp. *lactis* BPL1 (CECT8145) has potential therapeutic and/or preventive applications in MetS and diabetes-related disorders [192]. Probiotic species—*Saccharomyces boulardii*, *Lactobacillus acidophilus*, *Lactobacillus casei*, *Lactococcus lactis*, and *Lactobacillus reuteri*—have cell-free supernatants that exhibit antioxidant and anti-inflammatory qualities. These effects are initially observed in intestinal epithelial cells and then extend to the immune system. Not all probiotics demonstrate the same immunomodulatory effects; therefore, choosing the right strain for nutraceutical products requires careful consideration of several parameters. These probiotic-derived metabolites decreased the synthesis of IL-8 and prostaglandin-2 in human intestine HT-29 cell lines. The production of IL-1β, IL-6, TNF-α, and IL-10 by human macrophages is variably affected by probiotic supernatants, suggesting dose-dependent radical scavenging and anti-inflammatory properties [193]. The most economically significant bacterial group for the food sector is lactic acid bacteria (LAB), a subgroup of microorganisms that produce lactic acid. They provide a range of bioactives that are employed in formulating meals and drinks to increase the safety of fermented foods and prevent the growth of pathogenic bacteria; they also contain health-beneficial molecules, such as functional EPSs, enzymes, vitamins, peptides, anti-inflammatory agents, and antimicrobials [194]. By using antioxidant enzymes, including catalase, glutathione peroxidase, NADH-oxidase, and superoxide dismutase, LABs scavenge free radicals and reactive oxygen species, thereby fighting oxidative stress. These enzymes may help prevent certain diseases by lowering ROS levels. A 2,4,6-trinitrobenzene sulfonic acid (TNBS)-induced mouse model of Crohn’s disease has been used to investigate the anti-inflammatory effects of CAT or SOD-producing *Lactobacillus casei* strain BL23. Mice treated with BL23 recovered from initial weight loss more quickly, had higher intestinal enzyme activity, and experienced less intestinal inflammation than those not supplemented [195]. *L. acidophilus* 900, which has the most potent superoxide dismutase-like activity, and *L. plantarum* 30B, which has the highest catalase activity, were employed in an animal study. The findings indicated that for lowering intestinal inflammation, strains of *Lactobacillus* with dismutase-like activity were superior to those that produced catalase; this observation indicated that the mechanism of intestinal inflammation involves scavenging of superoxide anion radicals [196]. Peptides are among the postbiotics produced by the microbiota. Groups of effectors known as AMPs can deactivate microbes through membranolytic effects and nonlytic interactions with certain molecular targets [197]. Gram-positive species, *Lactococcus* and *Streptococcus*, synthesize nisin, a class I bacteriocin (lantibiotic), widely employed in biomedical science and as a food biopreservative. The proliferation of antibiotic-resistant bacterial species, such as methicillin-resistant *Staphylococcus aureus*, *Streptococcus pneumoniae*, *Enterococci*, and *Clostridium difficile*, is inhibited by nisin. It exhibits antimicrobial action against pathogenic Gram-positive and Gram-negative bacteria [198]. In a mouse model of inflammatory bowel illness caused by TNBS, *Lactobacillus plantarum* produced the Class II bacteriocin, plantaricin EF, which has anti-inflammatory properties. Mice exposed to *L. plantarum* NCIMB8826 did not show any significant levels of colonic TNF-α and IL-6. These findings point to a role for PlnEF production by *L. plantarum* in supporting digestive tract health [199].

### 6.6. Dietary Fiber as a Microbiota-Targeted Intervention

Sustaining health requires sufficient levels of dietary components such as fats, carbohydrates, proteins, vitamins, minerals, and fibers, the requirements of which vary according to age, gender, activity level, and individual health. Of them, dietary fibers are essential for maintaining a number of physiological processes [200]. Fruits, vegetables, whole grains, and legumes are the main sources of a variety of fiber types with a range of health advantages. Although numerous studies emphasize the broad ranging health advantages of dietary fibers, a substantial knowledge vacuum about the precise processes by which they affect various illnesses still exists [201]. Fibers—particularly those that ferment in the gut—help lower the chances of developing diabetes, control blood sugar levels, and enhance insulin sensitivity. In a similar vein, obesity, a worldwide epidemic impacting > 30% of the population, is associated with a higher risk of diabetes, cardiovascular disease, and other metabolic disorders [202]. Soluble dietary fibers affect T2DM indicators in mice models, by significantly increasing insulin sensitivity, which can delay the initiation of diabetes and help control blood sugar levels. Dietary fibers are also associated with a lower risk of weight gain, implying that they may mitigate certain adverse consequences linked to diets heavy in fat. Thus, these results suggest that soluble fibers may have greater advantages for metabolic health than other types. Diets rich in fiber and protein are consistently proven to optimize metabolic health and support weight regulation. This link was explicitly investigated in a study, which offered important insights into the interactions between various dietary components [203]; participants who received a healthy beverage with several ingredients and high in fiber and protein lost a significant amount of weight, as well as body fat and LDL cholesterol, two crucial markers of cardiovascular health. Adiponectin, a hormone essential for controlling glucose levels and fatty acid breakdown, were also significantly increased [203].

### 6.7. Microbiome Engineering and CRISPR-Based Therapeutic Approaches

Clustered Regularly Interspaced Short Palindromic Repeats (CRISPR) and CRISPR-associated proteins (Cas) modules function as adaptive immune systems in many bacteria and archaea. Microbes encode these defense systems which have a highly varied design and a rapid rate of mutation for *Cas* genes and distinct spacer content [204]. A guide (g)RNA that matches a target gene and the enzyme Cas9, which alters the genome via double-stranded DNA breaks, are the two essential components of the CRISPR/Cas9 gene-editing approach [205]. Studies on increasing the potential of gut microbiota-based medicines that show promise in treating a variety of illnesses and overcoming the associated obstacles are underway. CRISPR offers a unique way to treat illnesses related to gut bacteria by enabling specific alterations to induce particular effects. One approach is to modify the probiotic strain’s genome to provide novel functions or enhance the established benefits [206]. An additional strategy that requires fine-tuning is delivering the CRISPR system into the microbiota’s environment and altering the genomes of several bacterial phyla; this method, in which CRISPR functions inside the bowels, is called in situ gut microbiota alteration [207]. Since these novel treatments have only recently been introduced, it is vital to consider their possible long-term effects. As CRISPR allows precise genome editing in a variety of probiotic strains, it has substantially impacted the field of probiotic engineering.

CRISPR-Cas systems can be used to alter the natural functionalities of traditional LAB probiotics to offer stability [208]. Indoleamine 2,3-dioxygenase-1 (IDO1) is an enzyme involved in the immunoregulation of colorectal cancer. *Lactobacillus rhamnosus* GG (LGG)—a certified safe probiotic—was designed as a nanosystem using CRISPR/Cas9; these bacteria can enter the microenvironment of a gastrointestinal tumor to suppress IDO1 expression in tumor cells. This effect may recoup immunosuppression within the tumor microenvironment and cause immunogenic cell death after ROS production [178]. In another investigation on tumor therapy, *Escherichia coli* Nissle 1917 (EcN) was employed to deliver the CRISPR/Cas9 gene-editing system and immune agents directly to the cores of four T1 breast cancer cell lines. Non-pathogenic EcN bacteria were coated with polydopamine (pDA)—which can alter light into heat—to increase efficacy and safety. The coated microorganisms were then packaged in liposomes, which KO the *Hsp90α* gene in cancer cells by delivering CRISPR/Cas9 gene-editing tools. By depleting Hsp90α in the tumor microenvironment, this procedure, known as CRISPR-assisted photothermal-sensitized immunotherapy, lowered tumor cells’ heat resistance [209]. To boost butyrate synthesis, a study used CRISPR/Cas to alter the alcohol dehydrogenase (*aldh* and *adhE*) genes in the probiotic *Clostridium butyricum*, which produces ethanol. SCFAs such as butyrate mitigate a variety of physiological and pathological diseases and improve gut microbiota. Several methods for delivering the CRISPR/Cas system to microbial hosts have been refined. Hybrid plasmids and bacteriophages are frequently employed to alter the intestinal microbiome’s genomes. For example, temperate phages edited with CRISPR were designed to alter the microbiome’s bacterial composition and activity [210]. The use of CRISPR to create genetically modified probiotics has gained interest recently. Evidence supporting the therapeutic or preventive benefits of these novel probiotics is lacking, but may accumulate over time. For example, an animal study examined the impact of a CRISPR-engineered *Bacillus subtilis* probiotic termed BsS-RS06551 that produces butyrate, on obesity; biomass production was decreased by the KO of *skfa* and *sdpC*, which are involved in bacterial autolysis, to maximize the lifespan of BsS-RS06551 [211]. Butyrate synthesis improves production of the anti-inflammatory cytokine IL-10 but decreases body weight, insulin tolerance, and liver damage in obese mouse models. The modified strain of BsS-RS06551 showed encouraging outcomes for correcting obesity-related variables. The impacts of modified *E. coli* strains on phenylketonuria (PKU) were examined in a different study. PKU is a hereditary metabolic disease that can cause neurotoxicity due to high phenylalanine levels. The EcN strain lowers intestinal phenylalanine levels; it formed the foundation for a treatment plan. CRISPR was used to introduce three copies of *stlA*, which encodes phenylalanine ammonia-lyase (PAL), involved in phenylalanine conversion; additionally, it was also used to integrate two copies of *pheP* to improve EcN’s capacity to transport phenylalanine into the cell, by adding surface PAL from *P. luminescens*. The engineered probiotic obtained, TYS8500, transformed phenylalanine intracellularly and destroyed it extracellularly. When PKU mice were treated with TYS8500, their serum phenylalanine levels decreased, provides fresh avenues into the possibility of CRISPR-engineered probiotics to manage metabolic hereditary diseases [157].

## 7. Microbiome Modulation in Therapeutics: Drugs, Microbial Interactions, and Traditional Medicine

### 7.1. Pharmacological Variability and the Role of the Gut Microbiome

An important contributor to adverse drug responses (ADRs) and prolonged therapy, individual variability in drug response (IVDR) can have a significant negative impact on one’s health and finances. However, only a small percentage of IVDRs may be explained by genetic variations, despite substantial pharmacogenomics studies on the influence of an individual’s genetic background on pharmacokinetics (PK) and pharmacodynamics (PD). Recent research has focused on the role of gut microbiota—also referred to as the second genome and its metabolites—in influencing the effectiveness of treatments for human illnesses. As a result, the emerging field of “pharmacomicrobiomics” seeks to investigate the relationship between IVDRs or ADRs and variations in microbiota. Drug responses and gut microbiota liaisons that can influence PK—variations in drug absorption, distribution, metabolism, excretion, and plasma drug concentration dynamics—or PD—differences in drug targets or biological pathways that result in varying susceptibility to pharmacological effects—are referred to as pharmacomicrobiomics [212]. The Human Genome Project (HGP) was completed in 2003; it has fewer protein-coding genes, and in addition, genetic, epigenetic, and regulatory variants are insufficient to characterize IVDR phenotypically. As a result, it has limited applications in precision medicine. To comprehend the range of human phenotypic diversity, including its effects on immunological and medication response, and wellbeing, the emphasis has switched to the study of the gut microbiota and its composition, variation, and function. The gut microbiota is extremely mobile and adaptive compared to the human genome, like a cloud whose genetic pool and constituent parts are temporally and spatially unknown. Targeted alterations in gut microbiota affect drug response and disease pathogenesis, and may increase treatment efficacy, while reducing drug–drug interactions [213].

### 7.2. Drug–Microbiome Interactions in Clinical Therapeutics

Medications and xenobiotics can generate a variety of metabolic reactions, particularly in the gut microbiota, which can impact IVDR through direct effects on drug metabolism and toxicity, as well as host metabolic enzymes, transporters, and the immune system [92]. A potentially useful intermediary target for PK modulation that could eventually enhance clinical response is the existence of microbiota-encoded enzymes. A potent cytotoxic medication, MTX, is employed to treat autoimmune conditions like RA [214]. The gut microbiota is a significant factor affecting the body’s reaction to MTX. MTX may alter the gut microbiota’s composition by boosting the abundance of Lachnospiraceae and decreasing that of *Enterobacteria*, particularly *E. fecium* [208]. Notably, MTX and gut microbiota have a reciprocal relationship; the diversity of gut bacteria may impact responses to MTX therapy. Patients who had statistically elevated levels of *Prevotella maculosa* and a more diverse gut microbiota responded better to MTX [2]. ADRs are severe, and the response rate to MTX has a high IVDR, ranging from 10% to 80%, with only 40% of patients achieving a therapeutically relevant blood concentration, despite encouraging results [126]. However, data suggest that gut microbiota might be a significant factor in MTX-induced PD, and patient-specific treatment requires further research. In mice infected by *Mycobacterium tuberculosis*, the implications of anti-TB antibiotics on the composition of the gut microbiota have been investigated [28].

### 7.3. Microbiome-Based Therapeutic Strategies

The effectiveness of microbiome-focused therapy depends on dosage, composition, and treatment duration. Case studies reveal that probiotics are effective at 10^8^ to 10^11^ CFUs/day, with multi-strain formulas demonstrating greater and more consistent efficacy than single strains, especially for metabolic and inflammatory diseases. In most studies, treatment durations range from 4 to 12 weeks, though extended durations may be necessary for long-lasting benefits where gut microbes are reprogrammed, and chronic conditions are present [215].

A study in a mouse model suggested that three months of therapy with isoniazid (INH), rifampin (RIF), and pyrazinamide (PZA) resulted in a notable and long-lasting eco-dysbiosis. The gut microbiota’s composition changed, with Porphyromonadaceae, Bacteroidetes, and Proteobacteria growing and Clostridiales and *Lactobacillus* declining. Additionally, compared to the gut microbiota of mice without antibiotic treatment, a single dose of RIF reduced microbiome diversity, whereas INH or PZA alone altered microbiota composition. In particular, PZA enriched *Marvinbryantia*, but INH enriched *Gordonibacter* [13]. TB infection influenced the mucosal immune response, but did not significantly change the composition of the gut microbiota. The first-line anti-TB medication appears not to affect the gut microbiota in humans [141]. Anti-TB medications may also suppress the microbes required for intestinal homeostasis, which raises the risk of recurrent TB. Combining host route-targeting modifiers with anti-TB antibiotics may shorten treatment duration, suppress TB severity, and impede reinfection. Such a strategy may help in treating tuberculosis, according to recent research on host–pathogen correlations, host immunology, and host-targeted treatments. A drug, sulfasalazine (SAS), was first formulated to treat inflammatory diseases brought on by bacterial infections. However, it was found to be efficacious against ulcerative colitis (UC) [42]. SAS’s slow absorption via the upstream gastrointestinal tract is a unique quality. Instead, the gut microbiota in the colon breaks down SAS into its constituent parts. A PK investigation of healthy participants revealed that gut bacteria play a crucial role in SAS activation.

The effects of the microbiome donor and origin on the beneficial–detrimental proportions will reduce for treatments with higher levels of characterization and control (such as a single bacterial strain). Given the necessary characterization levels and reduced product complexity, the impact of the donor or origin of the microorganism(s) may become less significant in terms of risk when particular microbes are kept apart from recipients or other microbiome samples, like from food or the environment. However, h guidelines mandate that the origin of the microorganisms should constantly be clearly established [149]. The outcome of a stool preparation process appropriate for FMT is referred to as an “FMT product” in the USA. Since all FMT products are classified as biological products (drug products), they must submit an investigational new drug application and be approved by the FDA before being prescribed.

When the FMT product is not sourced from a stool bank and the other requirements outlined in the FDA guidelines—such as the patient’s consent as well as the screening and testing of the stool donor and stool sample—are fulfilled, there is an exception for the use of FMT to treat *C. difficile* infections that are non-responsive to standard therapies [216]. FMT can be administered as (a) a medical product or equivalent; (b) a therapeutic medication; (c) tissue and cell preparation; or (d) on a case-by-case basis, according to a recent study released by the Heads of Medicines Agencies [217]. The forthcoming EU “Regulation on standards of quality and safety for substances of human origin intended for human application” is likely to address this lack of uniformity regarding the regulatory status of FMT.

To more effectively approximate or represent physiological conditions, preclinical models, including organoids, gut-on-a-chip, and germ-free animal models, have been developed and used more frequently over the last decade. Additionally, these models can be used to determine the fundamental processes and cause-and-effect correlations in host–microbiome interactions. Human trials are necessary to determine the effectiveness of these therapies, even if preclinical models—such as animals, organoids, and gut-on-a-chip systems—are used for screening and preliminary testing of medicines [17]. Addressing the connections between changes in the microbiome and the development, progression, or worsening of diseases is the focus of causality in microbiome research. It entails determining if the microbiome composition is altered before a disease develops, during the course of illness or medication, or as separate elements that also play a role in the pathological process. This disparity is important because it clarifies the intricate relationship between microbial populations and host wellbeing. Furthermore, proving causation is necessary to pinpoint the molecular pathophysiology of diseases so that they can be the focus of therapeutic measures. Similarly, the invention and assessment of microbiome-based therapeutics depend on clarifying the causative involvement of the microbiome in disorder etiology [161]. Nevertheless, convincing and definitive evidence linking complex microbiomes with various inflammatory, metabolic, neoplastic, and neuro-behavioral problems is lacking. Furthermore, the reality that microbiomes are highly personalized populations of bacteria, viruses, fungi, protozoa, and archaea—which, along with their metabolites, may contribute to disease, either independently or in combination—makes it difficult to identify the causal elements of complex microbiomes that cause pathologies. Furthermore, certain components of the microbiome may prevent or lessen disease severity; their effects may vary depending on the situation and may not directly cause disease, but instead sensitize the host [126]. As a result, causal links are rarely straightforward but rather intricate and multifaceted, and may be impacted by mutually beneficial interactions within microbiomes.

### 7.4. Traditional Medicine, Diet, and Personalized Microbiome Modification

A proper diet, gastrointestinal function, and other aspects of lifestyle that inevitably impact gut health are of great significance in Ayurveda. Remarkably, some contemporary researchers have proposed that Ayurveda represents a type of conventional epigenetics [7]. Ayurvedic practitioners understood that every person has a distinctive psychophysiological profile, which is affected by variables such as dietary habits, digestive health, lifestyle, stress, and environment, even though they may not have been intrigued by the exact molecular pathways by which food might have regulated gene expression [218]. We are learning more about the connections between the microbiome and other Ayurvedic preventive and treatment methods thanks to modern science [219]. Āyurveda’s Tridoṣa paradigm serves as the basis for elucidating how the human brain and body function [220]. According to this notion, our body and mind are governed by three basic physiologic principles known as doṣas: Vāta, Pitta, and Kapha. The concept emphasizes the host and not the medical condition; it describes how various doṣas communicate and impact the manifestation and progression of a disorder [221]. Every doṣa is linked to characteristics unique to pathophysiology. In Āyurveda, Prakriti reflects the orderly constitution of the three doṣas at birth; the dominance of one doṣa over the others constitutes one of the fundamental phases in assessing a person’s health. Vikriti is defined as any departure from the Prakriti or natal doṣa distributions. Prakriti allocation involves phenotyping an individual based on their physical characteristics, dietary and bowel behaviors, immunity to illness, recuperation, mental abilities, metabolic rate, and other attributes [222]. Therefore, identifying unique microbial fingerprints peculiar to Prakriti is a promising avenue for tailored treatment [223]. These Āyurvedic therapeutic techniques are similar to contemporary medical treatment approaches that underscore dietary and lifestyle changes [224]. Modern medical science offers the potential to improve therapies according to an individual’s genetic and epigenetic patterns thanks to sophisticated “omic” tools. The Tridoṣa approach, in which Ayurvedic doctors and practitioners suggest customized treatment depending on a patient’s Prakriti, may be the Ayurvedic counterpart of precision medicine. Vāta, Pitta, and Kapha suggest unique microbiome compositions [225]. In Ayurveda, food is considered a medicine because it is thought that all ailments originate in the gut. An essential component of Ayurvedic treatments is a healthy diet. In addition to a wide range of medicinal herbs, Ayurveda uses several spices, herbs, mineral salts, and other natural products—many of which are common foods in and of themselves—to help treat particular ailments and restore and maintain physiological balance. Polyherbal or herb–mineral mixtures constitute the majority of Ayurvedic medications [226]. A multitude of formulations are successful against respiratory conditions, including asthma [227] and gut disorders such as IBS [228]. These formulations can also suppress the primary protease of SARS-CoV-2 [229]. Based on the idea of Shatkriyākalā, a flexible preventive therapy synchronized with phases of disease prediction (Shata = six, Kriyā = treatment choice, Kalā = phase of disease progression), Āyurveda suggests preventive medication. According to contemporary science, there are six phases of disease progression, including oxidative damage, immunological abnormalities, and anatomical and physiological alterations that contribute to disease onset and the development of anomalies.

Employing integrated therapy techniques that consider the season, dietary habits, lifestyle, and medications, Shatkriyākalā allows an avenue to address the advancement of every stage of a disease with appropriate and planned interventions. Ritucharya, or the seasonal behavioral regimen, is the Ayurvedic practice of adjusting one’s diet and lifestyle according to the season. It can control the gut microbiota, maintaining a person’s health throughout the year. The Ayurvedic concept of seasonal changes in dietary habits is essential, as different foods are suitable for each season. Consuming foods that are in season and grown nearby can help the body’s natural cycles, enhance general health and wellbeing, and lessen the negative effects of food production on the environment [230]. Furthermore, non-seasonal food could cause dysbiosis and other bodily imbalances [231]. A recent network pharmacology study has demonstrated the regulatory effects of Ritucharya-based food consumption on HIF-1, p53, PI3K-Akt, MAPK, cAMP, Ras, Wnt, NF-kappa B, IL-17, TNF, and cGMP-PKG-based signaling systems, as well as enhancing rehabilitation from COVID-19 infection.

It is becoming increasingly clear that dietary and lifestyle choices have a substantial impact on the beneficial microbial community in the human gut [232]. A disruption in this community can induce inflammatory conditions, pathogen susceptibility, and the current epidemic of metabolic health issues, including non-communicable diseases. The host could develop a season- and Prakriti-specific gut microbial composition as a defense against illnesses and infections by embracing Ritucharya, combining diet and lifestyle with seasonal fluctuations. Table 2 compiles Ayurvedic formulations traditionally used for gut health. The cited studies primarily report clinical outcomes or symptomatic improvement; however, direct microbiome profiling using sequencing-based approaches was limited or absent in most studies, highlighting the need for integrative microbiome–metabolome analyses in future investigations.

## 8. Limitations of This Study

This review has several limitations. Differences in microbiome assessment techniques, including variations in sequencing platforms and analytical pipelines, may contribute to heterogeneity across studies. Inconsistencies in study design, population characteristics, and experimental models may introduce bias and restrict direct comparisons. These methodological challenges must be considered when interpreting integrated findings. Numerous studies have demonstrated the significant influence of the human gut microbiota on health. One could classify dysbiosis as a medical issue. A disparity in the composition or function of the gut and non-gut-associated microbiomes is caused by systemic dysbiosis, which can manifest clinically from metabolic disorders to cancer. The term “normal gut microbiota” has not yet been defined precisely. Genetics, dietary choices, lifestyle, geography, and environmental factors differentially influence a person’s gut microbiota. Research on animals has contributed greatly to our understanding of dysbiosis. In addition, although Ayurvedic formulations are increasingly discussed in the context of microbiome modulation, well-designed, large-scale clinical trials evaluating standardized formulations, appropriate controls, and robust study designs remain limited. These methodological challenges must be considered when interpreting integrated findings.

## 9. Conclusions

Numerous investigations in humans and animals have produced substantial evidence of a direct correlation between diseases and microbiome imbalance. To enhance our comprehension of dysbiosis and the potential to prevent or treat illness, we must establish communication between the routes that comprise these interactions. The gut–organ axis refers to various connections that control gut–organ functional relationships. Gaining insights into the gut–organ–microbiota axis has improved our knowledge of several disease pathologies and provided opportunities to investigate microbial therapies through microbiome modulation. Diseases can be treated by targeting dysbiosis, which can be viewed as the cause, progression, or result of those ailments. Any area of the body with a microbiome unique to a person can be modulated. Depending on the characteristics of a disrupted ecosystem and underpinning genetic susceptibility to disease, dysbiosis may affect various hosts in multiple ways. Dysbiosis is influenced by metabolites synthesized by the gut microbiota, such as SCFA. The urine, hydrogen breath, intestinal permeability, and microbial diversity tests, thorough gastrointestinal waste assessment, and SCFA quantification are examinations used to detect dysbiosis. Numerous therapeutics, including drugs, alter the microbiome composition. To combat dysbiosis, dietary and lifestyle modifications must be considered as treatment measures. Given specific diets and lifestyles, the microbiome might change on a daily, weekly, or monthly basis. Disease development may be influenced by metabolites, such as SCFA, produced by the gut microbiota. Specific diets can impact numerous gut flora and metabolites. It is possible to affect the composition and operation of the gut microbiome by tackling dysbiosis with dietary and lifestyle modifications, such as shunning highly processed foods, which may be the most evident, non-invasive, and quick methods. In the realm of microbial therapies, studies and clinical trials are still underway. Diagnosing and treating dysbiosis can change many people’s lives. The methods used to repair dysbiotic conditions include dietary and lifestyle changes, gut antibiotic treatment, and, in more severe cases, fecal transplantation. Another strategy, exploring the traditional wisdom of healthy living, might resolve the “imbalance” conundrum. Potential approaches to treating gut–lung axis dysbiosis have been elucidated by the classical Indian medical system, Āyurveda, and its beliefs and concepts underlying gut health. A robust basis for preserving a healthy gut flora can be found in ancient Ayurvedic doctrines and customs of living in harmony with nature. We might discover the secret to optimal health and regulating our gut flora by returning to these roots.

## Figures and Tables

**Table 1:** Key bacterial strains and clinical implications of microbiome-related disorders.

Disorder	Associated Bacterial Stains	Alterations	Clinical Implications	References
Irritable Bowel Syndrome (IBS)	*Bifidobacterium* spp., Lactobacillus genera: Lacticaseibacillus, Limosilactobacillus, *Methanobrevibacter smithii*, *Escherichia coli*	Reduced Bifidobacterium and consortium of the reclassified Lactobacillus genera; elevated Methanobrevibacter and *E. coli*.	Higher gas production, altered motility, and inflammation.	[131]
Inflammatory Bowel Disease (IBD)	*Faecalibacterium prausnitzii*, *Roseburia* spp., *Eubacterium* spp., *E. coli*	Reduction in anti-inflammatory species (*F. prausnitzii*, Roseburia) overgrowth of pathogenic *E. coli*.	Loss of gut barrier integrity and chronic inflammation.	[132]
Clostridioides difficile Infection	*Clostridioides difficile*, reduced Bacteroidetes and Firmicutes diversity	Overgrowth due to disrupted microbiota (e.g., post-antibiotics).	Severe diarrhea and colitis.	[132]
*Helicobacter pylori* Infection	*Helicobacter pylori*	Colonizes the stomach lining and reduces protective microbial diversity.	Gastritis, ulcers, and increased gastric cancer risk	[133]
Colorectal Cancer	*Fusobacterium nucleatum*, *Bacteroides fragilis*, *Escherichia coli*	Enrichment of *F. nucleatum* and *B. fragilis*	Promotes tumorigenesis via inflammation and DNA damage.	[134]
Diverticulitis	*Bacteroides fragilis*, *Escherichia coli*, *Enterococcus* spp.	Altered microbial diversity and increased inflammation.	Pain, fever, and abscess formation.	[119]
Metabolic Disorders	*Akkermansia muciniphila*, *Bacteroidetes* spp., *Firmicutes* spp.	Reduced Akkermansia; altered Firmicutes/Bacteroidetes ratio	Obesity, insulin resistance, and increased inflammation.	[135]
Celiac Disease	*Bifidobacterium* spp., members of the reclassified Lactobacillus genera: Lacticaseibacillus, Limosilactobacillus), increased Enterobacteriaceae.	Reduced beneficial bacteria and increased pathogenic strains.	Triggers inflammatory responses in the gut.	[136]
Autism Spectrum Disorders	*Bacteroides* spp., *Clostridium* spp., *Prevotella* spp.	Decreased Prevotella; increased Clostridium.	Altered gut–brain axis signaling, behavioral symptoms.	[137]
Cardiovascular Diseases	Members of the reclassified Lactobacillus genera: Lacticaseibacillus, Limosilactobacillus, *Bifidobacterium* spp., Firmicutes	Increased trimethylamine-N-oxide (TMAO)-producing species.	Links to atherosclerosis and hypertension.	[138]

**Table 2:** Different Ayurvedic formulations for Gut health.

Ayurvedic Formulation	Pharmaceutical Form and Composition Type	Typical Dose and Duration of Administration	Evidence Base/Number of Patients	Therapeutic Area	References
Marichyadi Churna	Powder (Churna) and Polyherbal	1–5 g orally, twice daily after meals with warm water or buttermilk 2–4 weeks (traditional use); 14 days in reported clinical studies	Small clinical studies and classical texts; limited patient numbers.	IBS and digestive disorders	[233]
Takrarista	Fermented liquid (Arishta) and Polyherbals	10–30 mL orally, once or twice daily after meals 2–6 weeks (traditional practice)	Traditional use; controlled clinical trials with defined patient numbers are limited.	IBS	[234]
Pippalyasava	Fermented liquid (Asava) and Polyherbals	10–30 mL orally, twice daily after meals 3–6 weeks	Evidence largely based on classical literature and observations; patient numbers were not consistent.	Digestive disorders	[235]
Abhayarishta	Fermented liquid (Arishta) and Polyherbals	15–30 mL orally, twice daily after meals 2–6 weeks	Traditional and observational studies; limited standardized clinical trial data.	Digestive disorders	[236]
Dadimashtaka Churna	Powder (Churna) and Polyherbals	1–3 g orally, twice daily after meals 1–3 weeks	Classical Ayurvedic use; no large-scale clinical trials reported.	Diarrhea	[237]
Bhunimbadi Churna	Powder (Churna) and Polyherbals	1–5 g orally, twice daily after meals 2–4 weeks	Traditional usage supported by small clinical observations.	Diarrhea, malabsorption, and dysentery	[233]
Chitrakadi Vati	Tablet (Vati) and Herbominerals	250–500 mg orally, twice daily after meals 2–4 weeks	Small clinical and pharmacological studies; patient numbers vary.	Indigestion, constipation	[234]
Swadishta Virechana Churna	Powder (Churna) and Herbominerals	3–5 g orally, once daily (usually bedtime) Short-term use (1–2 weeks)	Traditional purgative formulation; limited modern clinical trials.	Detoxification and gut cleansing	[235]
Avipattikar Churna	Powder (Churna) and Herbominerals	1–3 g orally, twice daily after meals 2–4 weeks	Widely used; evidence mainly from classical texts and small clinical studies.	Gastritis, indigestion	[236]
Lavan Bhaskar Churna	Powder (Churna) and Herbominerals	1–2 g orally, twice daily after meals 2–3 weeks	Traditional use; controlled trials with reported sample sizes are limited.	Gastritis	[237]

## Data Availability

The raw data supporting the conclusions of this article will be made available by the authors on request.

## Abbreviations

The following abbreviations are used in this manuscript:

ADRs	Adverse drug responses
AMPs	Antimicrobial peptides
APCs	Antigen-presenting cells
BCAAs	Branched-chain amino acids
CAT	Catalase
CD	Cluster of differentiation
ChREBP	Carbohydrate response element binding protein
CVD	Cardiovascular diseases
DAMPs	Damage-associated molecular patterns
DCs	Dendritic cells
DiMDI	Dextran Sulfate Sodium-induced Microbial Dysbiosis Index
DNA	DNA
DNMTs	DNA methyltransferases
DSS	Dextran sulfate sodium
FDA	The Food and Drug Administration
FMT	Fecal microbiota transplantation
FXR	Farnesoid X receptor
GALT	Gut-associated lymphoid tissue
GI	Gastrointestinal
GPCRs	G protein-coupled receptors
H3K4me3	Histone H3 lysine 4 trimethylation
HDAC3	Histone deacetylase 3
HDACi	Histone deacetylase inhibition
HDL-c	High-density lipoprotein cholesterol
HFD	High-fat diet
HFDs	High-fat diets
HGP	Human Genome Project
IBD	Inflammatory bowel disease
IECs	Intestinal epithelial cells
IELs	Intraepithelial lymphocytes
IFNγ	Interferon gamma
IL	Interleukin
ILA	Indole-3-lactic acid
ILFs	Isolated lymphoid follicles
IRAKs	Interleukin-1 receptor-associated kinases
IVDR	Individual variability in drug response
LAB	Lactic acid bacteria
LBPs	Live biotherapeutic products
LPS	Lipopolysaccharides
MALT	Mucosa-associated lymphoid tissue
MAMPs	Microbial-associated molecular patterns
MET	Methionine
MetS	Metabolic syndrome
miRNA	MicroRNA
MTX	Methotrexate
MyD88	Myeloid differentiation primary response 88
NAFLD	Nonalcoholic fatty liver disease
ncRNAs	Non-coding RNAs
NGPs	Next-generation probiotics
PAMPs	Pathogen-associated molecular patterns
PCOS	Polycystic ovary syndrome
PD	Pharmacodynamics
PK	Pharmacokinetics
PKU	Phenylketonuria
PPIs	Proton pump inhibitors
PRRs	Pattern recognition receptors
PZA	Pyrazinamide
SAM	S-adenosylmethionine
SAS	Sulfasalazine
SCFAs	Short-chain fatty acids
SDI	Stroke Dysbiosis Index
SOD	Superoxide dismutase
SREBP-1	Sterol regulatory element binding protein 1
T2D	Type 2 diabetes mellitus
T2DM	Type 2 diabetes mellitus
TAAR1	Trace amine-associated receptor 1
TET2/3	Ten-Eleven translocation 2/3
TGR5	Takeda G-protein-coupled receptor 5
TIRAP	TIR domain-containing adaptor protein
TJs	Tight junctions
TLR4	Toll-like receptor 4
TLRs	Toll-like receptors
TMA	Trimethylamine
TMAO	Trimethylamine N-oxide
TNBS	2,4,6-trinitrobenzene sulfonic acid
TNF	Tumor necrosis factor
TNFα	Tumor necrosis factor alpha
VLDL	Very low-density lipoproteins
WT	Wild-type
